# Integrated *In Vitro* and *In Silico* Profiling of Piperazinyl Thiosemicarbazone Derivatives Against *Trypanosoma cruzi*: Stage-Specific Activity and Enzyme Inhibition

**DOI:** 10.3390/ph19010182

**Published:** 2026-01-20

**Authors:** Héctor A. Baldoni, María L. Sbaraglini, Darío E. Balcazar, Diego G. Arias, Sergio A. Guerrero, Catalina D. Alba Soto, Wioleta Cieslik, Marta Rogalska, Jaroslaw Polański, Ricardo D. Enriz, Josef Jampilek, Robert Musiol

**Affiliations:** 1 Facultad de Química, Bioquímica y Farmacia, Universidad Nacional de San Luis, Chacabuco 915, San Luis 5700, Argentina; 2 Instituto Multidisciplinario de Investigaciones Biológicas de San Luis (Consejo Nacional de Investigaciones Científicas y Técnicas), Universidad Nacional de San Luis, Av. Ejército de Los Andes 950, San Luis 5700, Argentina; 3 Laboratory of Bioactive Research and Development, Medicinal Chemistry, Department of Biological Sciences, Faculty of Exact Sciences, National University of La Plata, La Plata CP1900, Argentina; msbaraglini@wesleyan.edu; 4 Centro de Estudios Parasitológicos y Vectores, Consejo Nacional de Investigaciones Científicas y Técnicas, Universidad Nacional de La Plata, La Plata CP1900, Argentina; 5 Laboratorio de Enzimología Molecular, Instituto de Agrobiotecnología del Litoral, Santa Fe 3000, Argentina; 6 Facultad de Bioquímica y Ciencias Biológicas, Universidad Nacional del Litoral, Santa Fe 3000, Argentina; 7 Instituto de Microbiología y Parasitología Médica, Departamento de Microbiología, Facultad de Medicina, Universidad de Buenos Aires, Buenos Aires C1053, Argentina; 8 Institute of Chemistry, University of Silesia, 75 Pułku Piechoty 1a, 41-500 Chorzow, Poland; wioleta.cieslik@us.edu.pl (W.C.);; 9 Institute of Chemistry, University of Silesia, Szkolna 9, 40-007 Katowice, Poland; josef.jampilek@gmail.com; 10 Department of Chemical Biology, Faculty of Science, Palacky University Olomouc, Slechtitelu 27, 779 00 Olomouc, Czech Republic

**Keywords:** piperazinyl thiosemicarbazones, *Trypanosoma cruzi*, antitrypanosomal activity, cruzipain inhibition, structure-activity relationships

## Abstract

**Background:** *Trypanosoma cruzi*, the causative agent of Chagas disease, remains a major public health concern, and there is a continued need for new antitrypanosomal agents. Thiosemicarbazone (TSC) derivatives have emerged as a promising class of compounds with potential antiparasitic activity. **Objectives:** This study aimed to report the synthesis, characterization, and biological profiling of a novel series of thiosemicarbazone derivatives as antitrypanosomal agents against *Trypanosoma cruzi*. **Methods:** Fourteen new compounds and six previously described analogues were prepared and characterized by ^1^H/^13^C nuclear magnetic resonance (NMR) spectroscopy and mass spectrometry (MS). As a preliminary *in vitro* screen, activity was assessed by direct parasite counting in epimastigote and bloodstream trypomastigote forms, as tractable models of replicative and infective stages sharing core metabolic targets with intracellular amastigotes. Epimastigote potency was quantified as half-maximal effective concentrations (EC_50_) derived from dose–response curves, whereas trypomastigote response was evaluated as percent viability after treatment at a fixed concentration of 20 µM. Mechanistic profiling included inhibition assays against the cysteine protease cruzipain (CZP) and selected redox defense enzymes, complemented by *in silico* similarity clustering and binding-pose affinity scoring. **Results:** A nitro-methoxy-substituted TSC showed potent CZP inhibition but limited trypomastigote efficacy, whereas brominated analogues displayed dual-stage activity independent of CZP inhibition. Tanimoto similarity analysis identified distinct structure–activity clusters, linking nitro-methoxy substitution to epimastigote selectivity and brominated scaffolds to broader antiparasitic profiles, with hydrophobicity and steric complementarity as key determinants. Enzymatic assays revealed no significant inhibition of cytosolic tryparedoxin peroxidase (cTXNPx) or glutathione peroxidase type I (TcGPx-I), suggesting redox disruption is not a primary mode of action. *In vitro* and *in silico* analyses showed low or no non-specific cytotoxicity under the tested conditions, supporting further optimization of these derivatives as antitrypanosomal preliminary hits. Key hits included derivative **3e** (epimastigote EC_50_ = 0.36 ± 0.02 µM) and brominated analogues **2c** and **2e** (epimastigote EC_50_ = 3.92 ± 0.13 and 4.36 ± 0.10 µM, respectively), while docking supported favorable binding-pose affinity (e.g., ΔG*_S_*_-pose_ = −20.78 ± 2.47 kcal/mol for **3e**). **Conclusions**: These results support further optimization of the identified thiosemicarbazone derivatives as preliminary antitrypanosomal hits and provide insight into structure–activity relationships and potential mechanisms of action.

## 1. Introduction

Chagas disease (CD), or American trypanosomiasis, is a vector-borne parasitic disease caused by the flagellated protozoa *Trypanosoma cruzi* (*T. cruzi*), a member of the *Trypanosomatidae* family. It remains one of the most significant neglected tropical diseases in Latin America, with considerable socioeconomic impact. This zoonosis affects an estimated 7 million people and places over 100 million at risk, causing more than 10,000 deaths annually in endemic regions. Moreover, globalization has facilitated its spread to non-endemic countries, such as the United States and those in Western Europe, transforming CD into an emerging global health challenge [1,2].

The complex life cycle of *T. cruzi* alternates between two proliferative stages, epimastigotes in the insect vector and amastigotes in the mammalian host, and a non-proliferative, infective trypomastigote stage. Transmission occurs via triatomine bugs, after which the parasite invades host cells, differentiates into intracellular amastigotes, and replicates prior to transforming into bloodstream trypomastigotes that disseminate infection [3]. Clinically, Chagas disease progresses from an acute phase, often asymptomatic or with mild symptoms, through an indeterminate stage, to a chronic phase characterized by cardiac, gastrointestinal, or neurological complications [3,4]. In early antitrypanosomal drug discovery, rational screening cascades therefore prioritize *T. cruzi* epimastigotes and trypomastigotes as primary *in vitro* models. Epimastigotes provide a robust axenic system for detecting compounds with intrinsic trypanocidal activity against a proliferative stage, while bloodstream trypomastigotes represent the non-proliferative, infective form responsible for dissemination and tissue invasion. Both stages are experimentally tractable, share key metabolic and biochemical targets with intracellular amastigotes yet circumvent the confounding effects of host-cell permeability and metabolism scavenging, inherent to amastigote assays. Consequently, intracellular amastigotes are best reserved for secondary validation, following identification of preliminary hits with direct antiparasitic activity and acceptable safety profiles. Despite the considerable burden of morbidity and mortality associated with the disease, treatment options remain limited to benznidazole and nifurtimox, nitro-heterocyclic drugs introduced more than 50 years ago [5]. These treatments are effective during the acute phase and may help slow disease progression if given early. However, their efficacy declines in chronic infection. Moreover, the lengthy treatment courses, absence of child-friendly formulations, and frequent side effects, ranging from gastrointestinal disorders to skin reactions, often result in dose reductions or treatment discontinuation. This highlights the urgent need for new drugs that improve tolerability, shorten treatment duration, and act against both acute and chronic stages [6,7].

To address these limitations, research has increasingly focused on identifying parasite-specific molecular targets. Four principal targets have emerged: (1) ergosterol biosynthesis [8,9], (2) cysteine protease cruzipain (CZP) [10,11], (3) purine salvage pathways [12,13], and (4) pyrophosphate metabolism [14,15]. Among these, inhibition of ergosterol biosynthesis and CZP has been the most actively pursued [16,17].

CZP, a major lysosomal cysteine protease crucial for parasite survival and host cell invasion, has been targeted by multiple inhibitor classes that interact with its catalytic triad (Cys25, His162, and Asn182) [18,19]. Noteworthy examples include vinyl sulfone inhibitors [18], peptide vinyl sulfones [20], selenosemicarbazones [21,22], and amidines [23]. While many of these show potent CZP inhibition, this does not always correlate with antiparasitic activity.

Thiosemicarbazones (TSCs) constitute a class of covalent inhibitors capable of forming reversible tetrahedral adducts with the thiolate group of Cys25 via nucleophilic attack. Nonetheless, several TSC compounds exhibit antiparasitic activity independent of CZP inhibition [24,25,26]. TSCs are small, metal-chelating molecules with known antiviral and anticancer properties, attributed to their ionophoric behavior, redox activity, and reactive oxygen species generation [27]. The interest in TSCs as anti-*T. cruzi* agents date back to the 1970s, when Wilson et al. reported activity of arylaldehyde derivatives in both *in vitro* and *in vivo* models [28]. A major advance occurred in 2002, when McKerrow and colleagues, in collaboration with Parke-Davis, identified over 30 TSCs with CZP inhibitory effects [29].

**Figure 1:**
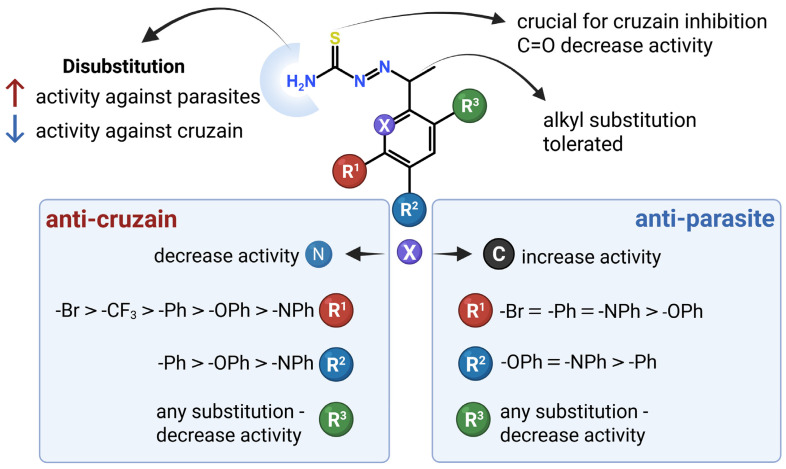
Schematic summary of aryl thiosemicarbazone structure-activity relationships relevant to *T. cruzi*, based on Ref. [25]. There are preferential substitutions on the aryl moiety that can strengthen cruzipain binding (including tolerance at the 2, 4, 5, and 6 positions, proposed to project toward the S2 specificity pocket) while also illustrating that several analogues exhibit strong antiparasitic activity despite lacking measurable cysteine protease inhibition, consistent with protease-independent activity pathways reported in the same study. Created in BioRender. Musiol, R. (2026). https://BioRender.com/tbjqrj5 (accessed on 15 January 2026).

In the context of CD, chemical modification of TSC scaffolds has led to selective anti-*T. cruzi* compounds, including some that act via mechanisms unrelated to CZP inhibition [30,31,32]. Previous SAR studies on aryl thiosemicarbazones revealed that subtle modifications at aryl-ring substituents can modulate cysteine protease engagement without directly dictating antiparasitic efficacy. Specifically, halogen substituents (e.g., Br, Cl) at the 3 and/or 5 positions enhance affinity for CZP by occupying the main S2 specificity pocket [25]. However, multiple analogues exhibit potent antiparasitic activity despite poor prote-ase inhibition, indicating that TSC scaffolds can operate through both protease-dependent and -independent mechanisms depending on substitution patterns [25]. These findings are schematically summarized in Figure 1 to frame our design rationale and contextualize the relationship between CZP inhibition and phenotypic activity in the new piperazinyl TSC series.

Our previous work on pyrazine-, piperazine-, and quinoline-based TSCs revealed strong antiproliferative effects, disruption of antioxidant defenses, apoptosis induction, and antifungal potential via iron chelation [33,34,35,36,37,38,39,40]. With these results in mind, we have developed a new series of thiosemicarbazone (TSC)-piperazinyl compounds based on a rational selection of features previously found to enhance antiparasitic activity. This new chemical series includes 20 new derivatives (**2**–**10**), which were synthesized as described elsewhere [33,34,35,36,37,38,39,40]. The aims of the present work were to (i) quantify *in vitro* activity against *T. cruzi* epimastigotes and trypomastigotes, (ii) benchmark preliminary host-cell safety in normal human dermal fibroblasts (NHDF), and (iii) explore potential mechanisms of action through inhibition assays against cruzipain (CZP) and redox enzymes (cTXNPx and TcGPx-I), complemented by covalent docking analysis. This integrated workflow was intended to prioritize the most promising chemotypes for subsequent optimization and more complex intracellular models.

## 2. Results and Discussion

### 2.1. Chemistry

**Scheme 1:**
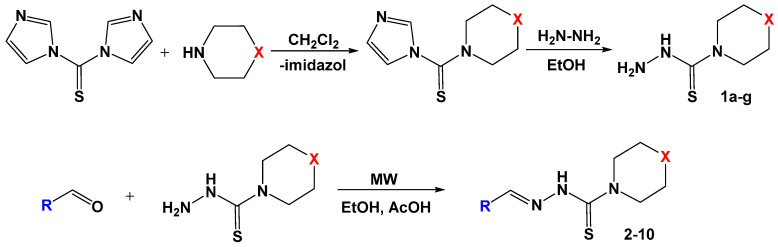
Synthesis of the thiosemicarbazides **1a**–**g** and thiosemicarbazone derivatives **2**–**10**. (i) (1,1′-thiocarbonyl)bis-1*H*-imidazole (5 mmol) and the appropriate piperazine/morpholine derivative (5 mmol) in CH_2_Cl_2_ (25 mL), stirred at room temperature for 24 h; aqueous extraction (3× H_2_O), drying (anhydrous MgSO_4_), filtration, and solvent removal. (ii) Hydrazine hydrate (5 mmol) in EtOH (25 mL), stirred at room temperature and then refluxed for 2 h; cooling to room temperature to precipitate; and recrystallization from MeOH to afford **1a**–**g**. (iii) Aldehyde/ketone (0.5 mmol) and thiosemicarbazide **1a**–**g** (0.5 mmol) in EtOH (5 mL) with glacial AcOH (two drops) under microwave irradiation (83 °C, 20 min; ≤50 W) in sealed glass tubes, followed by recrystallization from MeOH to afford **2**–**10**. The R and X denote the substituents shown in Figure 2.

The synthesis of thiosemicarbazones (TSCs) was carried out following established procedures [38]. The synthetic route used to obtain both the thiosemicarbazides and their corresponding TSC derivatives is outlined in Scheme 1.

The required thiosemicarbazide precursors (**1a**–**g**) were synthesized from commercially available reagents in a two-step sequence, as previously described [37,38]. In brief, equimolar (5 mmol) (1,1′-thiocarbonyl)bis-1*H*-imidazole and the appropriate piperazine or morpholine derivative were stirred in dichloromethane (25 mL, room temperature, 24 h), and the resulting thioketone intermediates were isolated after aqueous extraction, drying (anhydrous magnesium sulfate), filtration, and solvent removal.

**Figure 2:**
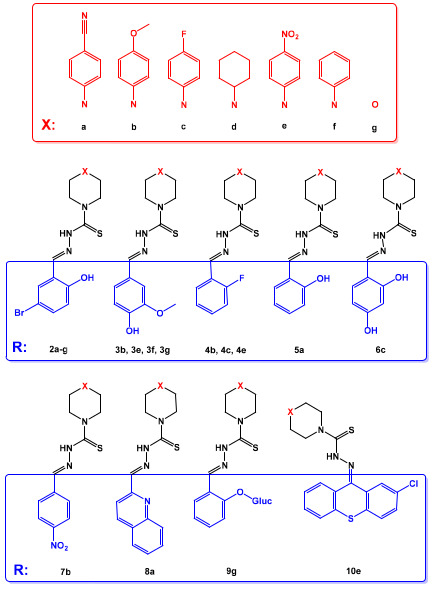
Structures of a new series of thiosemicarbazone derivatives **2**–**10**.

Subsequent treatment with hydrazine hydrate (5 mmol) in ethanol (25 mL; reflux, 2 h) afforded the N-substituted piperazine/morpholine-based thiosemicarbazides (**1a**–**g**) (Figure 2) after recrystallization from methanol, generally in high yields (59–97%) and with melting points consistent with literature reports (**1a** 86%, 179–180 °C; **1b** 81%, 194–195 °C; **1c** 97%, 180–181 °C; **1d** 59%, 178–179 °C; **1e** 87%, 207–208 °C; **1f** 69%, 173–174 °C; **1g** 89%, 160–161 °C).

The final TSC analogues (**2**–**10**) were synthesized via condensation of the corresponding aldehydes or ketones (0.5 mmol) with thiosemicarbazides **1a**–**g** (0.5 mmol) in ethanol (5 mL), catalyzed by glacial acetic acid (two drops), under microwave irradiation (83 °C, 20 min; reactor power not exceeding 50 W). This microwave-assisted methodology, previously optimized by our group [39], proved highly efficient, affording products in good to high yields (30–97%) and purity without the need for flash chromatography and enabling straightforward purification by methanol recrystallization.

All synthesized compounds were characterized by ^1^H and ^13^C NMR spectroscopy (Figures S1–S22) and mass spectrometry (MS). Across the TSC series, ^1^H NMR spectra consistently showed the diagnostic imine/azomethine proton (CH=N/CH) at δ 7.99–9.14 and a downfield thioamide NH at δ 11.02–11.75 (with the NH at δ 9.14 for **10e**), together with phenolic OH resonances at δ 8.94–11.68 where applicable; methoxy substituents appeared at δ 3.69–3.87 and piperazine/morpholine CH_2_ signals at δ 3.12–4.15. LC-MS measurements supported the assigned compositions (calcd/found, *m/z*): **2b** 449.06/449.04; **2c** 435.02/435.36; **2d** 425.10/425.10; **2e** 464.03/464.03; **2f** 419.05/419.03; **3b** 399.14/399.41; **3e** 414.12/414.17; **3f** 370.14/370.13; **3g** 294.09/294.09; **4b** 371.13/371.38; **4c** 361.12/361.01; **4e** 386.10/386.29; **9g** 428.14/427.98; **10e** 510.08/510.07.

### 2.2. Antitrypanosomal Activity

**Figure 3:**
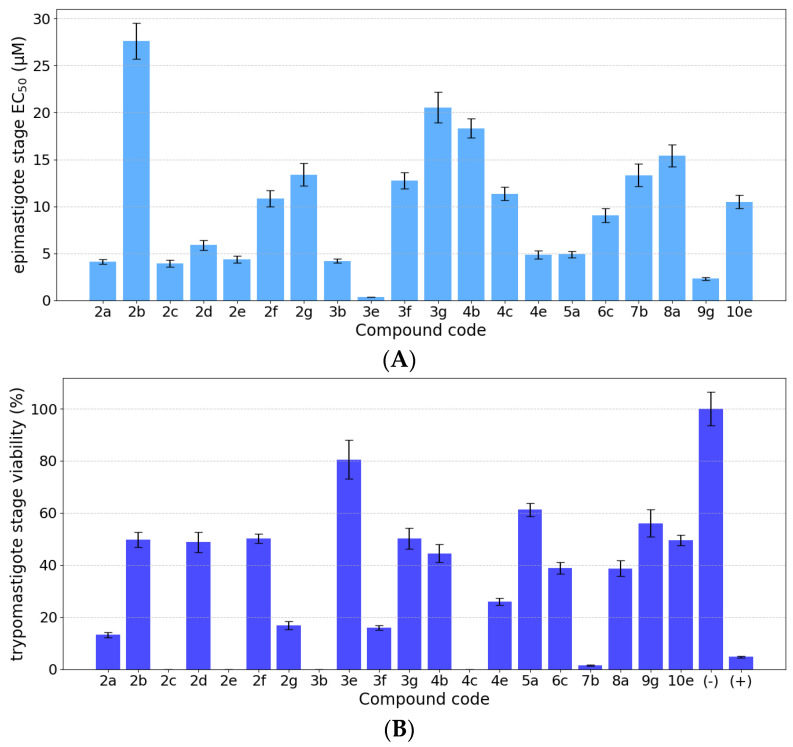
Comparison of *T. cruzi* parasite response to piperazinyl thiosemicarbazone derivatives **2**–**10**. Panel (**A**) epimastigote stage EC_50_ (μM) at day 4; panel (**B**) trypomastigote stage (derivatives at cc 20 μM). Negative control (-) DMSO, positive control (+) benznidazole.

To assess the trypanocidal potential of the synthesized compounds, *in vitro* assays were performed on both the epimastigote and trypomastigote forms of *T. cruzi*. The parasite viability in each case was determined by direct counting using a Neubauer hemocytometer chamber. For epimastigotes, the half-maximal effective concentration (EC_50_) was calculated from dose-response curves, providing a quantitative measure of the antiproliferative effect. In contrast, trypomastigote viability was evaluated by measuring the percentage of surviving parasites following treatment with each compound at a fixed concentration of 20 µM. The number of surviving parasites was monitored after exposure, and the results are presented in Figure 3 and Table 1 (see also Figure S23). Because trypomastigote viability was evaluated at a single concentration (20 µM) and a single exposure time (24 h), these data provide a screening-level ranking rather than a quantitative potency estimate; concentration-response profiling will be required to derive trypomastigote EC_50_ values for prioritized compounds.

The treatment of *T. cruzi* with the tested piperazinyl thiosemicarbazone derivatives resulted in marked reductions in parasite viability across both the epimastigote and trypomastigote stages. This observation is fully consistent with previous studies, which demonstrated that disubstitution at the -NH_2_ (R position in Scheme 1) within the thiosemicarbazone scaffold increases efficacy against parasites but lower cruzipain inhibition potency [25]. The EC_50_ values, calculated based on epimastigote viability, ranged from 0.36 µM for compound **3e** to 27.63 µM for compound **2b**, showing a considerable inhibitory effect on the proliferative form of the parasite (Figure 3A). Particularly noteworthy were compounds **2c**, **2e**, and **3b**, which exhibited EC_50_ values equal to or below 4.36 µM and achieved near-complete suppression of epimastigotes. These compounds also fully eliminated trypomastigote viability at 20 µM (Figure 3A,B), underscoring their potent trypanocidal activity against both life-cycle stages.

In contrast, trypomastigote viability exhibited greater variability, ranging from 0% to 80.5%. Notably, compound **3e** demonstrated paradoxical activity, showing exceptional inhibition of epimastigotes but poor suppression of trypomastigotes, with 80.5% of parasites remaining viable (Figure 3B). This divergence underscores the importance of assessing compound efficacy against both life stages, as the observed disparities may reflect stage-specific mechanisms of action or differential drug susceptibility.

Structural analysis revealed distinct contributions of specific substituents to trypanocidal activity. The presence of nitro (-NO_2_) groups, as seen in compounds **2e**, **3e**, **4e**, and **10e**, was associated with strong inhibition of epimastigotes (EC_50_ ≤ 10.48 µM), but produced inconsistent effects on trypomastigotes. For example, while **2e** completely suppressed trypomastigote viability (0%), **3e** and **4e** showed limited efficacy, with 80.5% and 25.9% viability, respectively. These results suggest that the brominated aryl core in the 2-series may enhance trypomastigote targeting more effectively than the methoxy- or fluorine-substituted scaffolds found in the 3- and 4-series.

Methoxy (-OCH_3_) substituents also demonstrated life stage-dependent effects on antiparasitic activity. Compound **3b** displayed potent efficacy against both parasite forms, with an EC_50_ of 4.19 µM for epimastigotes and complete elimination of trypomastigotes. In contrast, compound **2b** exhibited only moderate activity, with an EC_50_ of 27.63 µM and 49.7% trypomastigote viability. These results suggest that the antiparasitic potential of methoxy groups is strongly influenced by their structural environment. In particular, the presence of the bromophenol scaffold in **3b** appears to enhance the functional impact of the methoxy group, thereby maximizing efficacy across both life stages.

On the other hand, halogen substitutions also played a significant role in modulating antiparasitic activity. Bromine, consistently present in the 2-series compounds, was associated with strong inhibition of trypomastigote viability, ranging from 0% to 50.2%. Particularly, compounds **2c** and **2e** achieved complete suppression of trypomastigotes (0% viability), underscoring the effectiveness of brominated derivatives. In contrast, fluorine exhibited a clear positional effect. While compounds such as **2c** and **4c** demonstrated full trypomastigote inhibition, compound **6c** showed diminished activity (38.8% viability), likely due to differences in the fluorine substitution pattern on the aromatic ring. Overall, the 4-series derivatives, despite incorporating fluorine, were generally less effective, reinforcing the superior dual-stage efficacy of brominated analogues.

Notably, compound **9g**, which features a glycosyl moiety, exhibited pronounced inhibitory activity against epimastigotes (EC_50_ = 2.3 µM), yet only moderate suppression of trypomastigotes (56.08% viability). This disparity may reflect enhanced aqueous solubility or epimastigote-specific uptake mechanisms facilitated by the sugar group. In agreement with these observations, Fonseca et al. previously reported that sugar-containing thiosemicarbazones displayed low micromolar potency against *T. brucei*, but showed only modest activity against *T. cruzi* [41]. In contrast, compound **10e**, bearing a thiophene-chlorine scaffold, demonstrated moderate activity across both parasite stages (epimastigote EC_50_ = 10.48 µM, trypomastigote viability 49.5%), suggesting that its hybrid structure could serve as a template for the development of more balanced dual-stage inhibitors.

Although the 2-series compounds, such as **2c** and **2e**, emerged as the most promising, outliers like **8a**, a bicyclic derivative with modest activity (epimastigote EC_50_ = 15.39 µM; trypomastigote viability = 38.7%), illustrate that increased molecular complexity does not inherently translate into improved efficacy. Collectively, these findings delineate key structure-activity relationships: nitro and methoxy groups are associated with enhanced activity against epimastigotes, whereas bromine appears critical for achieving broad-spectrum potency. Moreover, these results are fully consistent with the findings of Greenbaum et al. [25], who identified Br-Phenyl, *O*-Phenyl, and *N*-Phenyl substituents at the 3′ or 4′ positions of the aryl ring (R in Scheme 1) as particularly favorable for binding the CZP target and for their antiparasitic activity, highlighting their potential for further development. However, the stage-specific activity differences observed in compounds such as **3e** and **9g** emphasize the need for life stage-informed strategies in antiparasitic drug design.

The present results derive from epimastigote (replicative form) and trypomastigote (non-replicative, infective form) assays and therefore provide a first-pass prioritization of chemotypes rather than definitive evidence of *in vivo* efficacy. Although the epimastigote model is a practical platform for initial screening, stage-specific differences were evident in this series, including compounds with strong epimastigote potency but limited trypomastigote killing, and conversely compounds that eliminated trypomastigotes at 20 µM despite only mid-micromolar epimastigote EC_50_ values. Such divergence indicates that activity against one life-cycle stage does not necessarily translate to others, and supports the use of complementary stage assays for triage. Importantly, intracellular amastigote assays are currently the gold standard for identifying drug candidates against Chagas disease, because they integrate host-cell penetration, intracellular exposure, and the clinically relevant replicative stage. Therefore, the absence of intracellular amastigote data limits direct translation of these findings and motivates follow-up studies in infected host-cell models for the most promising hits prioritized here.

### 2.3. Toxicity Results

Extensive experimental evidence has established that thiosemicarbazones are chemically versatile compounds with pronounced cytotoxic and anticancer effects [42,43]. In view of these well-documented toxicity mechanisms and their dependence on substitution pattern [44], metal-coordination behavior [45], and the nature of the targeted cell line [44,46], the toxicity profile of the synthesized compounds in this study was characterized by combining NHDF cytotoxicity assays with predictions estimated using machine-learning models for *in silico* toxicity endpoints. As shown in Table 2, a total of twenty compounds were evaluated.

Sixteen derivatives yielded reliable NHDF CC_50_ values, and four were not tested. *In silico*, all compounds were classified as inactive (non-cytotoxic), with P(inactive) scores between 0.56 and 0.70 at the default threshold of 0.5. Experimentally, thirteen derivatives, namely **2a**, **2g**, **3b**, **3e**, **3f**, **3g**, **4b**, **4c**, **4e**, **5a**, **7b**, **8a**, and **9g**, were non-cytotoxic (CC_50_ ≥ 25 µM), in agreement with the *in silico* prediction (true negatives). In contrast, three series-2 analogues, **2c** (14.4 ± 0.7 µM), **2d** (21.5 ± 3.0 µM), and **2e** (17.0 ± 3.7 µM), were cytotoxic to NHDF cells, despite being incorrectly predicted as inactive (false negatives). As depicted in Figure S24, the benchmark heatmap of *in silico* predictions against experimental NHDF cytotoxicity reveals a confusion matrix populated only with true negatives (TN) and false negatives (FN), with no true positives (TP) or false positives (FP) observed. Consequently, across the sixteen compounds with experimental data, the model achieved 81.3% overall accuracy, 0% sensitivity, 100% specificity, and an 81.3% negative predictive value, metrics entirely consistent with the absence of both TP and FP at the applied probability threshold.

The three false negatives share a common scaffold and cluster in the mid-micromolar CC_50_ range, suggesting an NHDF-specific toxicophore that is not captured by the *in silico* cytotoxicity model, which is primarily trained on hepatocyte and cancer cell-line data. Their moderate P(inactive) values (0.57–0.61) indicate low-confidence for non-cytotoxic assignments that nonetheless failed to trigger an alert. By contrast, all genuinely non-cytotoxic compounds (CC_50_ > 25 µM) were correctly identified as inactive, indicating that under the conditions tested the *in silico* prediction is conservative and more useful for flagging potential discrepancies than for positively identifying safe chemotypes. The four non-tested compounds, although predicted as inactive, could not be used for model evaluation.

To integrate antiparasitic potency with preliminary host-cell safety, an epimastigote selectivity index (SI) was calculated as the ratio of NHDF CC_50_ to epimastigote EC_50_ (SI = CC_50_/EC_50_) (Table 2), using the experimental cytotoxicity values in Table 2 and epimastigote EC_50_ values in Table 1. On this basis, compound **3e** displayed the highest SI lower bound (SI > 69), reflecting submicromolar epimastigote potency combined with no significant NHDF cytotoxicity up to 25 µM. Compound **9g** also showed a favorable SI lower bound (SI > 10.9). Among the dual-stage compounds that fully eliminated trypomastigotes at 20 µM, **3b** retained a favorable SI lower bound (SI > 6.0), whereas **2c** and **2e** showed lower SI values (~3.7–3.9) due to measurable NHDF cytotoxicity. These SI trends support prioritization of **3e** and **3b** as lead chemotypes for further optimization and expanded efficacy profiling. Overall, the data highlight both a structural limitation, namely series-2-specific toxicity in NHDF, and a methodological one, the use of a single probability cut-off on P(inactive) that is not tailored to scaffold- or cell-type-specific responses. These results therefore frame these derivatives as preliminary hits requiring optimization, rather than lead compounds ready for development.

### 2.4. Searching for Possible Mechanism of Action

Phenotypic efficacy across both parasite stages and preliminary toxicity assessment were prioritized as criteria for identifying preliminary hits. We therefore examined the TSC derivatives’ inhibitory effects on selected molecular targets, including CZP binding.

#### 2.4.1. Cruzipain Inhibition

**Figure 4:**
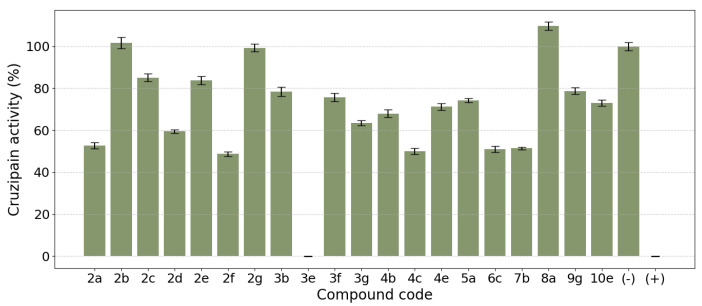
Percentage of cruzipain catalytic activity in response to piperazinyl thiosemicarbazone derivatives **2**–**10** (at ca. 100 μM). Negative control (-) DMSO, positive control (+) E64 inhibitor.

Cysteine protease CZP, a key enzymatic target particularly in *T. cruzi*, remains a central focus in the development of antitrypanosomal therapies due to its essential role in parasite survival and progression through its life cycle [47,48]. In this study, the activity of CZP was assessed across synthesized TSC derivatives, revealing notable differences in inhibitory efficacy. These results, summarized in Figure 4 and Table 1, underline the structural dependencies of CZP inhibition.

Among the compounds tested, derivative **3e** emerged as the most potent (0.0% residual activity), achieving complete inhibition of CZP catalytic activity. This pronounced effect is consistent with a highly productive alignment of the thiosemicarbazone pharmacophore within the active site, favoring formation and stabilization of a tetrahedral thioacyl adduct. In this context, the nitro substituent can increase the overall electron-withdrawing character of the aromatic system and strengthen polar interactions within the binding pocket, thereby promoting a tighter, more stabilized enzyme-inhibitor complex. In parallel, the methoxy group can modulate local electron density and conformational preferences while providing additional polar complementarity, which together may improve the probability of adopting a productive binding geometry. Concordantly, covalent docking predicts favorable thioacyl adduct poses for **3e** (Table 1), supporting a mechanistic interpretation in which the nitro and methoxy substituents jointly contribute to the strong CZP inhibition observed experimentally. It should be noted that nitro substituents are also well documented to influence antiparasitic activity through alternative mechanisms, namely strong electronic withdrawal and potential bioactivation pathways in kinetoplastids for nitroaromatics, though these are distinct from the covalent-CZP mechanism discussed herein. For instance, benznidazole activation against *T. cruzi* has been linked to parasite nitroreductase pathways and reactive metabolite formation [49]. In contrast, compound **8a** exhibited no inhibitory activity, highlighting the variability in compound efficacy across the series.

The 2-series derivatives, in general, demonstrated poor inhibitory activity. Most compounds in this series retained high levels of residual enzyme activity. For example, compounds **2b** and **2g** had negligible impact on CZP, while **2c** and **2e** showed only modest inhibition (14.8% and 16.2%, respectively). These findings suggest that bromine substitution does not inherently confer improved targeting of CZP, with the exception of **2a** and **2d**, which achieved moderate inhibition levels (47.3% and 40.5%, respectively).

Other derivatives showing moderate activity included **4c** and **6c**, with inhibition values of approximately 50%. In both cases, fluorine substitution appeared to partially disrupt enzyme function. Compound **9g**, which contains a glycosyl group, exhibited limited efficacy with only 21.3% inhibition, possibly due to steric hindrance or solubility issues that may interfere with proper binding to the active site.

Interestingly, compounds **3b** (21.6% inhibition) and **10e** (27.0% inhibition), previously identified as having strong trypanocidal activity, displayed weak inhibition of CZP. This suggests that their antiparasitic effects may be mediated through alternative mechanisms, such as interaction with other enzymatic pathways or cellular targets unrelated to CZP.

The 3-series of compounds yielded varied results. While **3e** showed exceptional activity, its analogues **3f** and **3g** produced only modest inhibition (24.3% and 36.4%, respectively). These observations underscore the importance of specific substituent combinations, such as the nitro and methoxy groups in **3e**, for optimal enzyme targeting. Similarly, derivatives from the 4-series, like **4e**, demonstrated limited effectiveness (28.8% inhibition), further reinforcing the structural complexity underpinning activity.

Taken together, these findings reveal a complex structure-activity relationship. While compound **3e** is a promising preliminary hit for CZP-targeted optimization, the lack of consistent correlation between CZP inhibition and trypanocidal potency, exemplified by **2e** and **3b**, underscores the need for broader mechanistic investigation. These observations are in line with previous studies [25], which showed that although certain substitutions on the aryl ring (i.e., R substituent in Scheme 1) improved affinity for cysteine proteases, many analogues without measurable protease inhibition still exhibited strong antiparasitic activity. In agreement with our findings, these authors also reported that halogens such as bromine and chlorine are well tolerated at the 3′- and/or 5′-positions of the aryl ring and are critical for cruzain inhibition. They proposed that the 3′-substituent of the aryl moiety at the TSC scaffold is positioned within the main specificity S2 pocket of the cruzipain protease [25]. Moreover, our results suggest that multiple mechanisms of action may be involved, including interactions with alternative molecular targets or protease-independent pathways. The identification of compounds with broad-spectrum antiparasitic activity further supports the existence of additional, as yet unidentified, biological targets [25].

#### 2.4.2. Antioxidant Defense Inhibition

The antioxidant defense system of *T. cruzi* plays a vital role in protecting the parasite against oxidative stress during host infection and its biological life cycle. This system relies on the coordinated function of two key enzymes: cytosolic tryparedoxin peroxidase (cTXNPx) and glutathione peroxidase-I (TcGPx-I). The cTXNPx enzyme utilizes the trypanothione system to reduce hydrogen peroxide and organic peroxides, while TcGPx-I detoxifies peroxides using glutathione (GSH) and tryparedoxin (TXN). Together, these enzymes help maintain redox homeostasis, ensuring parasite survival under hostile oxidative conditions [50,51,52].

**Figure 5:**
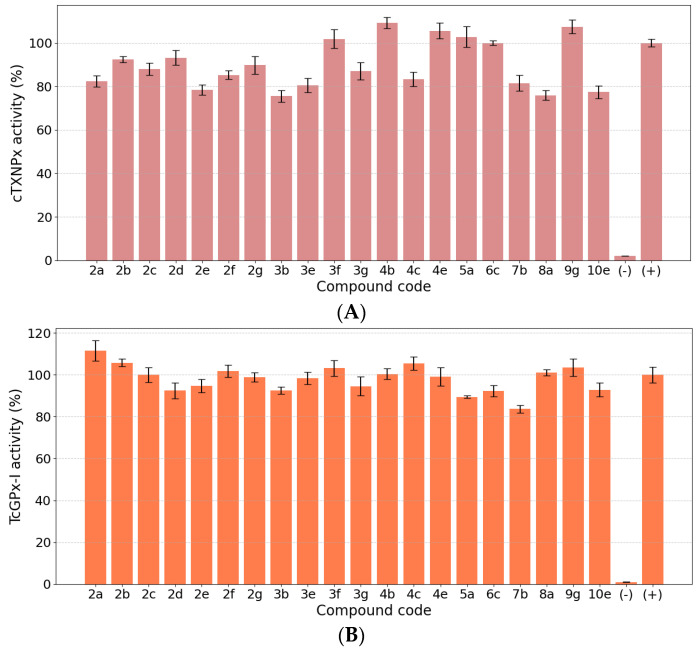
Percentage of redox defense enzyme activity remaining in response to piperazinyl thiosemicarbazone derivatives **2**–**10** (at cc 100 μM). Panel (**A**) cTXNPx; panel (**B**) TcGPx-I. Negative control (-) excluding test compounds and *t*-BuOOH, positive control (+) omitting test compounds.

To evaluate the potential of TSC derivatives as inhibitors of this redox detoxification system, their inhibitory effect on cTXNPx and TcGPx-I activity was assessed. The results, presented in Figure 5 and Table 1, reveal that most derivatives exhibited minimal inhibition, with residual enzyme activities typically exceeding 75% of control levels. For example, cTXNPx activity remained largely intact in the presence of compounds **2b**, **2c**, and **2d**, which preserved 92–93% of enzyme function. Compounds **2a** and **2f** also maintained high levels of activity, at 82% and 85%, respectively. A similar trend was observed for TcGPx-I, where compounds such as **2a**, **2f**, and **3f** not only retained activity but in some cases produced slight increases.

These data suggest that the antioxidant functions of cTXNPx and TcGPx-I remain largely unaffected by the tested derivatives. A few exceptions were noted: compound **2e** reduced cTXNPx activity to 78%, and **3b** to 75%, while **7b** decreased TcGPx-I activity to 84%. However, these inhibitory effects were modest and lacked consistency across the series, failing to reveal a discernible structure-activity relationship. Intriguingly, certain compounds such as **4e** and **9g** increased cTXNPx activity to 105% and 107%, respectively, further highlighting the variable nature of enzyme response.

Moreover, compounds **8a** and **10e**, despite demonstrating moderate trypanocidal effects, exhibited little impact on either enzyme, with residual activities ranging from 76% to 93%. This dissociation between enzyme inhibition and antiparasitic efficacy suggests that interference with the redox detoxification system is not the primary mechanism of action for these compounds.

These findings indicate that the trypanocidal activity of the TSC derivatives is unlikely to be mediated by disruption of cTXNPx or TcGPx-I. The preserved activity of both enzymes, even in the presence of active compounds, supports the hypothesis that alternative pathways or molecular targets are responsible for the observed antiparasitic effects.

### 2.5. Molecular Modeling

Biophysical assays for on- and off-target binding are low throughput, resource-intensive, and prone to interference and specificity issues [53], making them premature at these preliminary hit stage. Molecular modeling was therefore used to prioritize primary scaffolds, deferring experimental target validation as a deliberate strategic choice.

Several crystal structures detailing complexes between CZP and its inhibitors have been resolved and deposited within the Protein Data Bank, providing critical structural insight [54,55,56]. This structural data serves as an essential foundation for guiding subsequent molecular modeling efforts. Consequently, the availability of these structures has enabled reliable pose prediction and accurate calculation of binding free energy (ΔG_binding_) values specifically for the tetrahedral thioacyl adducts formed during the interaction between CZP and each of the twenty evaluated TSC derivatives. Moreover, as previously demonstrated in related studies [57,58], the methodology employed here has proven effective in delivering consistent and accurate results, even across diverse systems. Its application in the present work reflects its robustness and suitability for modelling covalent inhibition mechanisms.

**Figure 6:**
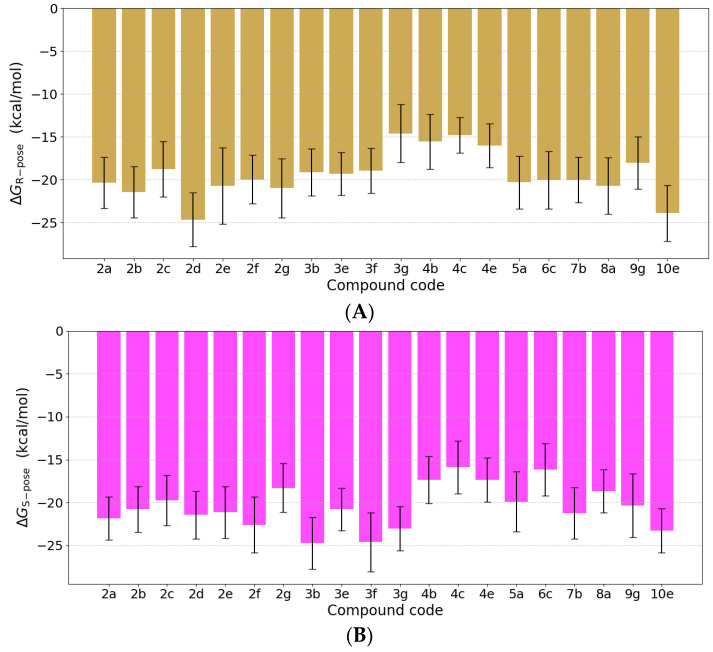
Calculated binding free energy (ΔG, in kcal/mol) for the tetrahedral thioacyl adducts formed through the interaction between cruzipain and thiosemicarbazone derivatives **2**–**10**. Panel (**A**) *R*-pose; and Panel (**B**) *S*-pose.

As shown in Figure 6 and detailed in Table 1, the resulting ΔG_binding_ values reveal considerable variation between the *R*- and *S*-binding poses, demonstrating significant stereoselectivity in the covalent binding of TSC derivatives to CZP.

In the **2a**–**2g** series, the electronic nature of para-substituents on the benzyl ring exerted a clear influence on binding pose preference. Derivatives with electron-withdrawing groups such as cyano (**2a**) and nitro (**2e**) favored the *S*-binding pose (ΔG*_S_* = −21.83 kcal/mol and −21.14 kcal/mol, respectively), both showing stronger binding affinities in the *S*-pose than in the *R*- (ΔG*_R_* = −20.35 and −20.73 kcal/mol). Conversely, compound **2b**, featuring a para-methoxy group, demonstrated a reverse preference for the *R*-pose (ΔG*_R_* = −21.43 kcal/mol vs. ΔG*_S_* = −20.79 kcal/mol), suggesting stabilization through potential polar contacts or altered π-stacking interactions mediated by the methoxy oxygen lone pairs. This observation illustrates how subtle electronic tuning can invert stereoselective trends within closely related analogues.

Interestingly, compound **2f**, lacking any para-substitution, showed the highest *S*-pose affinity within the series (ΔG*_S_* = −22.60 kcal/mol). This may indicate an optimal fit and reduced steric clash of the unsubstituted phenyl ring within the CZP active site.

Derivatives from the **3b**–**3g** series consistently exhibited higher *S*-pose affinities compared to the 2-series. Notably, compound **3b** had the most favorable *S*-binding free energy (ΔG*_S_* = −24.72 kcal/mol), followed closely by **3f** (ΔG*_S_* = −24.61 kcal/mol), suggesting that methoxy substitution on both aromatic rings synergistically enhances *S*-pose stabilization. However, **3g** displayed markedly reduced *R*-pose affinity (ΔG*_R_* = −14.61 kcal/mol), implicating the morpholine ring as a destabilizing moiety in the *R*-pose, likely due to steric or torsional constraints.

Compound **10e** presented nearly isoenergetic *S*- and *R*-binding poses (ΔG*_S_* = −23.27 kcal/mol; ΔG*_R_* = −23.92 kcal/mol), possibly reflecting a less stereochemically demanding interaction profile conferred by its chlorothiophene moiety.

The fluorinated derivatives from the 4-series showed notably weaker binding. For example, **4c** and **4e** exhibited *S*-pose ΔG values of −15.88 and −17.37 kcal/mol, respectively, suggesting that fluorine substitution may impair binding via electronic repulsion or poor complementarity within the pocket. Similarly, **5a** displayed lower affinity, underscoring the sensitivity of the binding pocket to structural modifications.

The stereochemical preferences of other derivatives further emphasize the complexity of CZP-TSC interactions. Compound **6c** strongly favored the *R*-pose (ΔG*_R_* = −20.06 kcal/mol vs. ΔG*_S_* = −16.18 kcal/mol), as did **8a** (ΔG*_R_* = −20.73 kcal/mol vs. ΔG*_S_* = −18.67 kcal/mol), suggesting that elongated conjugated systems may preferentially adopt *R*-binding configurations. Despite bearing a carbohydrate moiety, compound **9g** exhibited relatively low *R*-pose affinity (ΔG*_R_* = −18.02 kcal/mol), likely due to conformational restrictions imposed by the bulky sugar group.

Altogether, these binding free energy profiles point to the presence of stereo- and substituent-dependent interactions that dictate the binding behavior of TSC derivatives with CZP. These results support the need for careful stereochemical and electronic optimization in the design of more potent and selective CZP inhibitors.

### 2.6. Structure-Activity Relationship

The synthesized TSC derivatives exhibited distinct structure-activity relationships (SAR) in their inhibition of CZP and antitrypanosomal activity. Compound **3e** emerged as the most effective CZP inhibitor, with a null residual activity score that correlated with strong suppression of epimastigotes (EC_50_ = 0.36 μM). However, its limited effect on trypomastigotes (80.51% viability) highlights a stage-specific profile, suggesting that CZP modulation may be relevant in epimastigotes, whereas resistance mechanisms or target inaccessibility likely limit efficacy in the trypomastigote stage.

**Figure 7:**
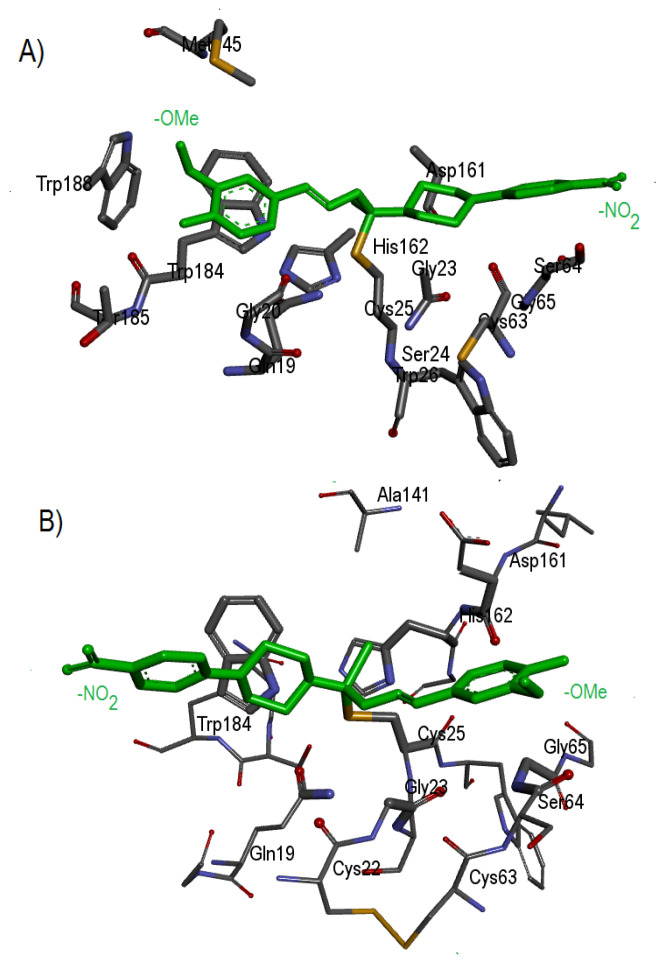
Binding pose of piperazinyl thiosemicarbazone derivative **3e** (green sticks) in the *R*- (**A**) and *S*- (**B**) tetrahedral thioacyl adducts within the cruzipain catalytic cleft. Residues within 4 Å from the ligand are shown.

Molecular modeling supports this, showing that nitro (-NO_2_) and methoxy (-OCH_3_) substituents synergistically enhance *S*-pose binding (ΔG*_S_* = −20.78 kcal/mol vs. ΔG*_R_* = −19.33 kcal/mol) (Table 1, Figure 7). Despite its potency, **3e** did not inhibit redox enzymes cTXNPx or TcGPx-I (with 80% and 98% residual activity, respectively), reinforcing that the redox defense mechanism is not involved in the antiparasitic results.

In contrast, brominated 2-series compounds **2c** and **2e** showed dual-stage activity, completely eliminating trypomastigotes (0% viability) and nearly eradicating epimastigotes (EC_50_ ≤ 4.36 μM). These results highlight the critical role of bromine substitution in enhancing trypomastigote targeting, a feature absent in methoxy- (3-series) or fluorine-substituted (4-series) analogues. For example, **3b** (a methoxy-only analogue of **3e**) displayed dual-stage inhibition (epimastigote EC_50_ = 4.19 μM, trypomastigote viability 0%), whereas **2b** (methoxy-substituted but lacking bromine) exhibited weaker activity (epimastigote EC_50_ = 27.63 μM, trypomastigote viability 49.72%). This suggests that bromine bulk or electronic character enhances interactions with trypomastigote-specific targets. Interestingly, both **2c** and **2e** were active despite minimal CZP inhibition (85.1% and 82% residual activity, respectively), implicating non-CZP pathways such as prodrug activation or metabolic interference.

Halogenation trends also proved substituent-dependent: fluorine enhanced activity in **2c** and **4c** (both with 0% trypomastigote viability), but not in **6c** (38.8% viability), likely due to variation in ring substitution patterns. Similarly, **9g**, containing a glycosyl moiety, effectively inhibited epimastigotes (EC_50_ = 2.3 μM) but had limited effect on trypomastigotes (56.0%), suggesting improved solubility or uptake in epimastigotes without a corresponding parasiticidal effect.

Compound **10e**, bearing a chlorothiophene scaffold, displayed moderate dual-stage activity (epimastigote EC_50_ = 10.48 μM, trypomastigote viability 49.5%) despite comparable *S*- and *R*-pose affinities (ΔG*_S_* = −23.27; ΔG*_R_* = −23.92 kcal/mol), reaffirming that steric and electronic complementarity, rather than binding free energy alone, governs target engagement.

Although the thiourea core present in all TSC facilitates target interaction, substituent effects primarily dictated activity. For instance, compound **2d**, with a bulky cyclohexyl group, showed reduced trypomastigote lethality (48.75% viability) despite moderate CZP binding (ΔG*_S_* = −21.45 kcal/mol), underscoring steric hindrance. In contrast, **7b**, with an unsubstituted phenyl ring, achieved near-complete trypomastigote eradication (1.5% viability) despite weak CZP inhibition (51.35% residual activity), suggesting alternative parasiticidal targets.

A general disconnect between CZP inhibition and antiparasitic efficacy was observed. For example, **2f**, with 48.65% CZP activity, showed limited trypomastigote suppression (50.2% viability), while **8a**, a bicyclic derivative, exhibited modest activity (epimastigote EC_50_ = 15.39 μM; trypomastigote viability 38.7%) with little effect on CZP. Additionally, cTXNPx and TcGPx-I remained largely unaffected across active compounds (>75% residual activity), eliminating redox disruption as a primary mechanism.

In summary, brominated 2-series derivatives (e.g., **2c**, **2e**) are the most promising dual-stage preliminary hits, while **3e** represents a promising scaffold for CZP-focused optimization. These findings support a stage-specific strategy, where substituents such as bromine or nitro modulate target engagement and selectivity across the parasite life cycle.

### 2.7. Tanimoto Similarity and Pharmacophore Correlations

**Figure 8:**
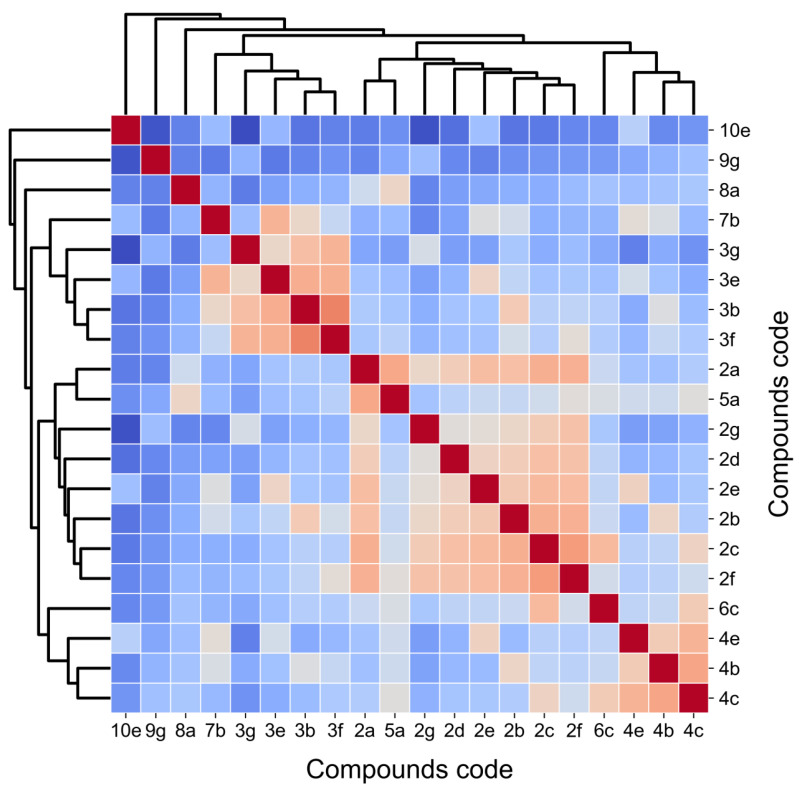
Heat map and hierarchical clustering of thiosemicarbazone derivatives **2**–**10**. Chemical similarity measured with the MACCS fingerprint and the Tanimoto coefficient. The mean intrasimilarity for derivatives is 0.49.

The Tanimoto similarity analysis (Ta) (Figure 8 and Table S1) provided quantitative validation of the above SAR trends and underlined the role of structural complexities in governing biological effects [59,60]. Clusters with high similarity (Ta score > 0.7) frequently reflected shared activity profiles, while exceptions underscored the dominant influence of specific substituents.

Compound **3e** (self-similarity, Ta = 1.0), featuring a nitro-methoxy pharmacophore, was predicted to be the most potent CZP inhibitor, consistent with its strong *S*-pose binding affinity (ΔG*_S_* = −20.78 kcal/mol). However, it displayed stage-specific activity, showing near-complete inhibition of the epimastigote stage (EC_50_ = 0.36 μM) but limited trypomastigote efficacy (80.51% viability), suggesting a CZP-independent mechanisms. Structurally related analogues such as **7b** (Ta = 0.75 with **3e**) exhibited comparable CZP inhibition (51.35% activity) but markedly enhanced trypomastigote elimination (1.5% viability), likely due to increased hydrophobicity from its unsubstituted phenyl ring. In contrast, compound **4e** (Ta = 0.576 with **3e**), bearing a fluorophenyl group, showed reduced enzymatic inhibition (71.19% residual activity), whereas derivative **10e** (Ta = 0.43 with **3e**), incorporating a sterically bulky chlorothiophene moiety, demonstrated moderate dual-stage activity despite favorable *S*-pose binding (ΔG*_S_* = −23.27 kcal/mol). These findings emphasize that thermodynamic stability alone is insufficient without steric complementarity.

The brominated derivatives **2c** and **2e** (self-similarity Ta = 1.0 each) exemplified the role of halogenation in dual-stage efficacy, eliminating trypomastigotes entirely (0% viability) and suppressing epimastigotes to EC_50_ ≤4.36 µM. Their broad activity contrasted with that of the fluorine-substituted analogues: compound **4c** (Ta = 0.492 with **2c**) displayed reduced epimastigote suppression (EC_50_ = 11.35 μM) but retained trypomastigote lethality, whereas **6c** (Ta = 0.731 with **2c**), bearing a difluorophenyl group, showed diminished trypomastigote stage activity (38.84% viability). The distinctive electronic and steric properties of bromine appeared critical in directing trypomastigote-specific pathways. Compounds **2c** and **2e** were particularly effective while maintaining substantial CZP activity (>80% residual), suggesting additional mechanisms such as prodrug activation.

Methoxy-substituted derivatives further demonstrated how electronic effects modulate activity. Compounds **3b** (self-similarity, Ta = 1.0) and **3f** (Ta = 0.846 with **3b**) exhibited weak CZP inhibition (78.38% and 75.68% residual activity, respectively), likely due to the electron-donating nature of the methoxy group diminishing reactivity with the catalytic Cys25 residue. Hybrid scaffolds, such as **5a** (Ta = 0.774 with **2a**) and **8a** (Ta = 0.655 with **5a**), illustrated how substitution patterns influence enzyme compatibility. The hydroxyl group in compound **5a** enabled moderate inhibition of CZP, resulting in 74.32% residual activity, whereas the fused quinoline scaffold in compound **8a** introduced steric hindrance, leading to a loss of inhibition (109.6% residual activity). In contrast, compound **9g** (Ta = 0.42 with **3g**), bearing a glycosyl moiety, exhibited strong epimastigote inhibition (EC_50_ = 2.30 μM) through non-enzymatic mechanisms, despite its poor binding free energy (ΔG_binding_ = −18.02 kcal/mol).

Outliers such as **2f** (Ta = 0.8 with **2c**) and **10e** underscored the mechanistic complexity involved. Despite partial CZP inhibition (48.65% residual activity), **2f** showed limited trypomastigote lethality (50.2% viability), possibly due to compensatory survival pathways. Similarly, the conformational rigidity of the chlorothiophene moiety in **10e** hindered CZP binding despite favorable thermodynamics, emphasizing the need to optimize stereoelectronic balance.

Taken together, the Tanimoto similarity analysis supported the SAR findings. The nitro-methoxy motif (e.g., **3e**, **7b**) was linked to CZP-dependent epimastigote viability, while bromination (e.g., **2c**, **2e**) contributed to dual-stage efficacy. Bulky substituents, such as the cyclohexyl group in **2d** and the glycosyl moiety in **9g**, were identified as sources of steric hindrance or solubility issues that potentially restrict enzyme binding. Furthermore, the preferred *S*-pose in **3e** underscored the importance of stereochemical precision.

## 3. Materials and Methods

### 3.1. Reagents and Instruments

All reagents were purchased from Sigma-Aldrich^®^ and used without further purification. Microwave reactions were carried out in a CEM reactor model DISCOVER^®^ BenchMateTM, in sealed 10 mL glass tubes. TLC experiments were performed on alumina-backed silica gel 40 F_254_ plates (Merck, Darmstadt, Germany). The plates were illuminated under UV (254 and 365 nm). The melting points were determined on an Optimelt MPA-100 apparatus (SRS, Stanford CA). All ^1^H and ^13^C NMR spectra were recorded on a Bruker Advance III 400 MHz FT-NMR spectrometer (400 MHz for ^1^H and 101 MHz for ^13^C, Bruker Comp., Karlsruhe, Germany). Chemical shifts are reported in ppm (δ) using the signal of the solvent (DMSO-*d_6_*) as the reference against the internal standard Si(CH_3_)_4_. Easily exchangeable signals were omitted when they were diffuse. Signals are designated as follows: s, singlet; d, doublet; dd, doublet of doublets; ddd, doublet of doublet of doublets; t, triplet; tt, triplet of triplets; td, triplet of doublets; m, multiplet. Mass spectra were measured using a LTQ Orbitrap Hybrid Mass Spectrometer (Thermo Electron Corporation, USA) with direct injection into an APCI source (400 °C) in the positive or negative mode.

### 3.2. Synthesis

The compounds were prepared in the following manner:

#### 3.2.1. General Procedure for Synthesizing Thiosemicarbazides (As Previously Described in [38])

Equimolar amounts (5 mmol) of (1,1′-thiocarbonyl)bis-1*H*-imidazole and the appropriate piperazine or morpholine derivative were dissolved in dichloromethane (25 mL) and stirred at room temperature for 24 h. The resulting mixtures were extracted three times with distilled water, and the combined organic layers were dried over anhydrous magnesium sulfate, filtered, and concentrated under reduced pressure using a rotary evaporator. The resulting thioketone intermediates were subsequently added to a solution of hydrazine hydrate (5 mmol) in ethanol (25 mL) at room temperature. The mixture was refluxed for 2 h, and then cooled to room temperature, resulting in the formation of a precipitate. The crude products were collected by filtration and purified by recrystallization from methanol to afford the final thiosemicarbazide derivatives.

*4-(4-Cyanophenyl)piperazine-1-carbothiohydrazide* (**1a**): White powder; yield 86%; mp: 179–180 °C (previously described in [38]).

*4-(4-Methoxyphenyl)piperazine-1-carbothiohydrazide* (**1b**): White powder; yield 81%; mp: 194–195 °C (previously described in [38]).

*4-(4-Fluorophenyl)piperazine-1-carbothiohydrazide* (**1c**): Light pink powder; yield 97%; mp: 180–181 °C (previously described in [38]).

*4-Cyclohexylpiperazine-1-carbothiohydrazide* (**1d**): White powder; yield 59%; mp: 178–179 °C (previously described in [37]).

*4-(4-Nitrophenyl)piperazine-1-carbothiohydrazide* (**1e**): Yellow powder; yield 87%; mp: 207–208 °C (previously described in [35,37]).

*4-Phenylpiperazine-1-carbothiohydrazide* (**1f**): White powder; yield 69%; mp: 173–174 °C (previously described in [38]).

*Morpholine-4-carbothiohydrazide* (**1g**): White crystal powder; yield 89%; mp: 160–161 °C (previously described in [37]).

#### 3.2.2. General Procedure for Synthesizing TSC (As Previously Described in [38])

To a solution of the thiosemicarbazide (0.5 mmol) and the appropriate aldehyde or ketone (0.5 mmol) in ethanol (5 mL), two drops of glacial acetic acid were added as a catalyst. The reaction mixtures were transferred to sealed glass tubes and subjected to microwave irradiation at 83 °C for 20 min, with the reactor power not exceeding 50 W. Upon completion, the resulting products were purified by recrystallization from methanol.

*N′-[(5-Bromo-2-hydroxyphenyl)methylidene]-4-(4-cyanophenyl)piperazine-1-carbothiohydrazide* (**2a**): Yellow powder; yield: 77%; mp: 218–219 °C (previously described in [36]).

*N′-[(5-Bromo-2-hydroxyphenyl)methylidene]-4-(4-methoxyphenyl)piperazine-1-carbothiohydrazide* (**2b**): Yellow powder; yield: 97%; mp: 203–204 °C; ^1^H-NMR (400 MHz, DMSO-*d_6_*, ppm): δ 3.12 (s, 4H, CH_2_), 3.70 (s, 3H, CH_3_), 4.07 (s, 4H, CH_2_), 6.90–6.82 (m, 3H, Ar-H), 6.95 (d, 2H, *J* = 8.9 Hz, Ar-H), 7.41 (dd, 1H, *J1* = 8.7 Hz, *J2* = 1.9 Hz, Ar-H), 7.68 (s, 1H, Ar-H), 8.44 (s, 1H, CH=N), 11.68 (s, 1H, NH, OH) (Figure S1); ^13^C-NMR (101 MHz, DMSO-*d_6_*, ppm): δ 49.0; 50.0; 55.7; 110.5; 114.8; 118.2; 119.5; 121.3; 131.9; 133.6; 135.9; 144.7; 145.3; 153.8; 156.7 (Figure S2); LC-MS: calculated for C_18_H_20_BrN_5_O_2_S [M+H]^−^ 449.06522 *m/z*, found 449.04718 *m/z*.

*N′-[(5-Bromo-2-hydroxyphenyl)methylidene]-4-(4-fluorophenyl)piperazine-1-carbothiohydrazide* (**2c**): Beige powder; yield: 76%; mp: 205–206 °C; ^1^H-NMR (400 MHz, DMSO-*d_6_*, ppm): δ 3.20 (s, 4H, CH_2_), 4.08 (s, 4H, CH_2_), 6.88 (d, 1H, *J* = 8.7 Hz, Ar-H), 7.00 (m, 2H, Ar-H), 7.08 (t, 2H, *J* = 8.7 Hz, Ar-H), 7.41 (dd, 1H, *J1* = 8.7 Hz, *J2* = 1.6 Hz, Ar-H), 7.68 (d, 1H, *J* = 1.3 Hz, Ar-H), 8.44 (s, 1H, CH=N), 11.67 (s, 1H, OH), 11.70 (s, 1H, NH) (Figure S3); ^13^C-NMR (126 MHz, DMSO-*d_6_*, ppm): δ 48.8; 49.1; 110.5; 115.9; 117.9; 119.2; 121.3; 131.9; 133.6; 144.7; 147.8; 155.8; 156.7; 157.7 (Figure S4); LC-MS: calculated for C_17_H_17_BrFN_5_OS [M-H]^−^ 435.0285 *m/z*, found 435.36 *m/z*.

*N′-[(5-Bromo-2-hydroxyphenyl)methylidene]-4-cyclohexylpiperazine-1-carbothiohydrazide* (**2d**): Yellow powder; yield: 76%; mp: 197–198 °C; ^1^H-NMR (400 MHz, DMSO-*d_6_*, ppm): δ 1.13–1.01 (m, 1H, CH), 1.19 (dd, 4H, *J1* = 19.6 Hz, *J2* = 9.8 Hz, CH_2_), 1.58 (d, 1H, *J* = 11.6 Hz, CH), 1.75 (d, 4H, *J* = 9.0 Hz), 2.29 (s, 1H, CH_2_), 2.57 (s, 4H, CH_2_), 3.89 (s, 4H, CH_2_), 6.86 (d, 1H, *J* = 8.7 Hz, Ar-H), 7.39 (m, 1H, Ar-H), 7.65 (t, 1H, *J* = 4.6 Hz, Ar-H), 8.40 (s, 1H, CH=N), 8.94 (s, 1H, OH), 11.75 (s, 1H, NH); ^13^C-NMR (126 MHz, DMSO-*d_6_*, ppm): δ 25.7; 26.3; 28.7; 31.2; 48.7; 65.9; 110.5; 119.3; 121.3; 131.9; 133.5; 144.5; 156.6; 179.7; LC-MS: calculated for C_18_H_25_BrFN_4_OS [M+H]^+^ 425.10051 *m/z*, found 425.1002 *m/z*.

*N′-[(5-Bromo-2-hydroxyphenyl)methylidene]-4-(4-nitrophenyl)piperazine-1-carbothiohydrazide* (**2e**): Orange powder; yield: 78%; mp: 224–225 °C; ^1^H-NMR (400 MHz, DMSO-*d_6_*, ppm): δ 3.68 (s, 4H, CH_2_), 4.12 (s, 4H, CH_2_), 6.88 (d, 1H, *J* = 8.7 Hz, Ar-H), 6.99 (d, 2H, *J* = 8.9 Hz, Ar-H), 7.41 (d, 1H, *J* = 8.4 Hz, Ar-H), 7.68 (s, 1H, Ar-H), 8.09 (d, 2H, *J* = 8.8 Hz, Ar-H), 8.46 (s, 1H, CH=N), 11.67 (s, 2H, NH, OH) (Figure S5); ^13^C-NMR (101 MHz, DMSO-*d_6_*, ppm): δ 45.6; 47.9; 110.6; 112.5; 119.3; 121.3; 126.2; 131.9; 133.6; 137.3; 144.9; 154.5; 156.7; 179.8 (Figure S6); LC-MS: calculated for C_18_H_18_BrN_5_O_3_S [M+H]^+^ 464.038641 *m/z*, found 464.0399 *m/z*.

*N′-[(5-Bromo-2-hydroxyphenyl)methylidene]-4-phenylpiperazine-1-carbothiohydrazide* (**2f**): White powder; yield: 72%; mp: 188–189 °C; ^1^H-NMR (400 MHz, DMSO-*d_6_*, ppm): δ 3.27 (s, 4H, CH_2_), 4.08 (s, 4H, CH_2_), 6.82 (t, 1H, *J* = 7.2 Hz, Ar-H), 6.88 (d, 1H, *J* = 8.8 Hz, Ar-H), 6.97 (t, 2H, *J* = 7.3 Hz, Ar-H), 7.25 (t, 2H, *J* = 7.8 Hz, Ar-H), 7.41 (dd, 1H, *J1* = 8.7 Hz, *J2* = 2.1 Hz, Ar-H), 7.68 (d, 1H, *J* = 2.1 Hz, Ar-H), 8.44 (s, 1H, CH=N), 11.67 (s, 1H, OH), 11.70 (s, 1H, NH) (Figure S7); ^13^C-NMR (126 MHz, DMSO-*d_6_*, ppm): δ 48.3; 51.2; 110.5; 116.2; 119.3; 119.7; 121.3; 129.5; 131.9; 133.6; 144.7; 150.9; 156.7; 179.8 (Figure S8); LC-MS: calculated for C_18_H_19_BrN_4_OS [M+H]^+^ 419.05356 *m/z*, found 419.03650 *m/z*.

*N′-[(5-Bromo-2-hydroxyphenyl)methylidene]morpholine-4-carbothiohydrazide* (**2g**): White powder; yield: 51%; mp: 197–198 °C (previously described in [36]).

*N′-[(4-Hydroxy-3-methoxyphenyl)methylidene]-4-(4-methoxyphenyl)piperazine-1-carbothiohydrazide* (**3b**): White powder; yield: 72%; mp: 174–175 °C; ^1^H-NMR (400 MHz, DMSO-*d_6_*, ppm): δ 3.16 (s, 4H, CH_2_), 3.69 (s, 3H, CH_3_), 3.82 (s, 3H, CH_3_), 4.04 (s, 4H, CH_2_), 6.83 (m, 2H, Ar-H), 6.94 (d, 2H, *J* = 9.0 Hz, Ar-H), 7.02 (d, 2H, *J* = 8.1 Hz, Ar-H), 7.23 (s, 1H, Ar-H), 8.02 (s, 1H, CH=N), 9.53 (s, 1H, OH), 11.10 (s, 1H, NH) (Figure S9); ^13^C-NMR (101 MHz, DMSO-*d_6_*, ppm): δ 50.2; 50.5; 55.7; 55.9; 109.4; 114.8; 116.0; 118.1; 122.0; 126.2; 144.6; 145.4; 148.5; 149.2; 153.7; 180.9 (Figure S10); LC-MS: calculated for C_20_H_24_N_4_O_3_S [M-H]^−^ 399.14963 *m/z*, [M-H]^−^ found 399.41 *m/z*.

*N′-[(4-Hydroxy-3-methoxyphenyl)methylidene]-4-(4-nitrophenyl)piperazine-1-carbothiohydrazide* (**3e**): Dark-yellow powder; yield: 52%; mp: 183–184 °C; ^1^H-NMR (400 MHz, DMSO-*d_6_*, ppm): δ 3.69–3.64 (m, 4H, CH_2_), 3.87 (s, 3H, CH_3_), 4.11–4.06 (m, 4H, CH_2_), 7.00–6.94 (m, 3H, Ar-H), 7.22 (s, 1H, Ar-H), 8.12–8.03 (m, 3H, Ar-H), 9.14 (s, 1H, CH=N), 9.52 (s, 1H, OH), 11.16 (s, 1H, NH) (Figure S11); ^13^C-NMR (101 MHz, DMSO-*d_6_*, ppm): δ 45.7; 46.6; 56.0; 112.5; 116.0; 120.3; 122.0; 126.3; 129.6; 137.2; 138.0; 148.5; 154.6; 181.0; 182.8 (Figure S12); LC-MS: calculated for C_19_H_21_N_5_O_4_S [M-H]^−^ 414.12415 *m/z*, found 414.17114 *m/z*.

*N′-[(4-Hydroxy-3-methoxyphenyl)methylidene]-4-phenylpiperazine-1-carbothiohydrazide* (**3f**): Yellow powder; yield: 43%; mp: 134–135 °C; ^1^H-NMR (500 MHz, DMSO-*d_6_*, ppm): δ 3.22 (s, 3H, CH_3_), 3.29 (t, 4H, *J* = 5.2 Hz), 4.05 (t, 4H, *J* = 5.1 Hz), 6.81 (m, 2H, Ar-H), 6.99 (m, 3H, Ar-H), 7.24 (m, 3H, Ar-H), 8.03 (s, 1H, CH), 9.54 (s, 1H, OH), 11.11 (s, 1H, NH); ^13^C-NMR (126 MHz, DMSO-*d_6_*, ppm): δ 48.5; 50.4; 55.9; 109.4; 115.9; 116.0; 119.7; 122.0; 126.1; 129.5; 144.6; 148.5; 149.2; 151.0; 180.8; LC-MS: calculated for C_19_H_22_N_4_O_2_S [M]^−^ 370.14689 *m/z*, found 370.13385 *m/z*.

*N′-[(4-Hydroxy-3-methoxyphenyl)methylidene]morpholine-4-carbothiohydrazide* (**3g**) Yellow powder; yield: 38%; mp: 169–170 °C; ^1^H-NMR (400 MHz, DMSO-*d_6_*, ppm): δ 3.68 (m, 4H, CH_2_), 3.80 (s, 3H, CH_3_), 3.89 (m, 4H, CH_2_), 6.81 (d, 1H, *J* = 8.1 Hz, Ar-H), 7.01 (d, 1H, *J* = 8.1 Hz, Ar-H), 7.19 (s, 1H, Ar-H), 7.99 (s, 1H, CH), 9.51 (s, 1H, OH), 11.08 (s, 1H, NH) (Figure S13); ^13^C-NMR (126 MHz, DMSO-*d_6_*, ppm): δ 51.4; 55.9; 64.5; 109.3; 116.0; 122.0; 126.1; 144.6; 148.5; 149.2; 181.1 (Figure S14); LC-MS: calculated for [M-H]^−^ C_13_H_17_N_3_O_3_S 294.09178 *m/z*, found 294.09143 *m/z*.

*N′-[(2-Fluorophenyl)methylidene]-4-(4-methoxyphenyl)piperazine-1-carbothiohydrazide* (**4b**): Yellow powder; yield: 30%; mp: 168–169 °C; ^1^H-NMR (400 MHz, DMSO-*d_6_*, ppm): δ 3.13 (s, 4H, CH_2_), 3.70 (s, 3H, CH_3_), 4.07 (s, 4H, CH_2_), 6.85 (d, 2H, *J* = 8.9 Hz), 6.95 (d, 2H, *J* = 9.0 Hz), 7.31–7.25 (m, 2H, Ar-H), 7.50–7.42 (m, 1H, Ar-H), 7.85 (t, 1H, *J* = 7.5 Hz, Ar-H), 8.40 (s, 1H, CH=N), 11.38 (s, 1H, NH) (Figure S15); ^13^C-NMR (126 MHz, DMSO-*d_6_*, ppm): δ 50.1; 50.3; 55.6; 114.8; 116.6; 118.2; 122.4; 125.4; 126.7; 132.1; 137.1; 145.4; 153.7; 160.1; 162.1; 181.0 (Figure S16); LC-MS: calculated for C_19_H_21_FN_4_OS [M-H]^+^ 371.13364 *m/z*, found 371.38 *m/z*.

*N′-[(2-Fluorophenyl)methylidene]-4-(4-fluorophenyl)piperazine-1-carbothiohydrazide* (**4c**): Yellow powder; yield: 87%; mp: 163–164 °C; ^1^H-NMR (400 MHz, DMSO-*d_6_*, ppm): δ 3.25–3.19 (m, 4H, CH_2_), 4.11–4.03 (m, 4H, CH_2_), 7.00–6.97 (m, 2H, Ar-H), 7.08 (t, 2H, *J* = 8.4 Hz, Ar-H), 7.32–7.25 (m, 2H, Ar-H), 7.50–7.42 (m, 1H, Ar-H), 7.85 (t, 1H, *J* = 7.5 Hz, Ar-H), 8.40 (s, 1H, CH=N), 11.40 (s, 1H, NH) (Figure S17); ^13^C-NMR (101 MHz, DMSO-*d_6_*, ppm): δ 49.4; 50.2; 115.7; 116.4; 117.8; 122.4; 125.4; 126.7; 132.1; 137.2; 147.9; 155.5; 157.9; 159.9; 162.3; 181.2 (Figure S18); LC-MS: calculated for C_18_H_18_F_2_N_4_S [M+H]^+^ 361.12923 *m/z*, found 361.01505 *m/z*.

*N′-[(2-Fluorophenyl)methylidene]-4-(4-nitrophenyl)piperazine-1-carbothiohydrazide* (**4e**): Dark-yellow powder; yield: 64%; mp: 182–183 °C; ^1^H-NMR (400 MHz, DMSO-*d_6_*, ppm): δ 3.70–3.65 (m, 4H, CH_2_), 4.15–4.08 (m, 4H, CH_2_), 6.99 (d, 2H, *J* = 9.4 Hz, Ar-H), 7.32–7.25 (m, 2H, Ar-H), 7.46 (q, 1H, *J* = 6.2 Hz, Ar-H), 7.88 (t, 1H, *J* = 7.3 Hz), 8.12–8.08 (m, 2H, Ar-H), 8.41 (s, 1H, CH=N), 11.43 (s, 1H, NH) (Figure S19); ^13^C-NMR (126 MHz, DMSO-*d_6_*, ppm): δ 45.8; 49.4; 112.5; 116.5; 120.2; 122.4; 126.3; 129.5; 132.1; 137.2; 154.6; 160.1; 162.1; 180.9 (Figure S20); LC-MS: calculated for C_18_H_18_FN_5_O_2_S [M-H]^−^ 386.10925 *m/z*, found 386.29 *m/z*.

*N′-[(2-Hydroxyphenyl)methylidene]-4-(4-cyanophenyl)piperazine-1-carbothiohydrazide* (**5a**): Yellow powder; yield: 73%; mp: 215–216 °C (previously described in [36]).

*N′-[(2,4-Dihydroxyphenyl)methylidene]-4-(4-fluorophenyl)piperazine-1-carbothiohydrazide* (**6c**): Yellow powder; yield: 57%; mp: 205–206 °C (previously described in [36,40]).

*N′-[(4-Nitrophenyl)methylidene]-4-(4-methoxyphenyl)piperazine-1-carbothiohydrazide* (**7b**): Orange powder; yield: 85%; mp: 180–181 °C (previously described in [37]).

*N′-[(Quinolin-2-yl)methylidene]-4-(4-cyanophenyl)piperazine-1-carbothiohydrazide* (**8a**): Yellow powder; yield 70%; mp: 202–203 °C (previously described in [35]).

*N′-2{[(3,4,5-Trihydroxy-6-hydroxymethylene)tetrahydropyran-2-yloxy]benzylidene}morpholine-4-carbothiohydrazide* (**9g**): Yellow powder; yield: 49%; mp: 188–189 °C; ^1^H-NMR (400 MHz, DMSO-*d_6_*, ppm): δ 3.20–3.16 (m, 1H, CH_2_), 3.70–3.65 (m, 4H, CH_2_), 3.95–3.90 (m, 4H, CH_2_), 4.57 (t, 1H, *J* = 5.7 Hz), 4.91 (d, 1H, *J* = 6.7 Hz), 5.04 (d, 1H, *J* = 5.3 Hz), 5.15 (dd, 2H, *J* = 13.1, 3.8 Hz), 7.07 (t, 1H, *J* = 7.5 Hz), 7.22 (d, 1H, *J* = 8.4 Hz), 7.38 (t, 1H, *J* = 7.8 Hz), 7.75 (d, 1H, *J* = 7.7 Hz), 8.56 (s, 1H, CH=N), 11.02 (s, 1H, NH) (Figure S21); ^13^C-NMR (101 MHz, DMSO-*d_6_*, ppm): δ 51.0; 61.1; 66.5; 70.1; 74.0; 76.8; 77.6; 101.7; 116.3; 122.8; 123.9; 125.6; 131.6; 141.0; 156.6; 181.1 (Figure S22); LC-MS: calculated for C_18_H_25_N_3_O_7_S [M+H]^+^ 428.14860 *m/z*, found 427.98267 *m/z*.

*N′-[2-Chlorothioxanthen-9-ylidene]-4-(4-nitrophenyl)piperazine-1-carbothiohydrazide* (**10e**): Orange powder; yield: 87%; mp: 177–178 °C; ^1^H-NMR (400 MHz, DMSO-*d_6_*, ppm): δ 3.61–3.51 (m, 4H, CH_2_), 3.95–3.86 (m, 4H, CH_2_), 6.96 (d, 2H, *J* = 9.2 Hz, Ar-H), 7.66–7.55 (m, 1H, Ar-H), 7.89–7.77 (m, 3H, Ar-H), 7.92 (d, 1H, *J* = 8.6 Hz), 8.13–8.00 (m, 2H, Ar-H), 8.37 (d, 1H, *J* = 2.0 Hz), 8.46 (d, 1H, *J* = 8.1 Hz), 9.14 (s, 1H, NH); ^13^C-NMR (126 MHz, DMSO-*d_6_*, ppm): δ 45.6; 46.6; 112.5; 126.2; 127.2; 127.6; 128.5; 129.3; 129.6; 130.0; 132.1; 133.3; 133.8; 135.9; 136.8; 137.2; 138.1; 154.2; 178.3; 182.7; LC-MS: calculated for C_24_H_20_ClN_5_O_2_S_2_ [M+H]^+^ 510.081969 *m/z*, found 510.0721 *m/z*.

### 3.3. Antitrypanosomal Activity Assays

#### 3.3.1. Epimastigote Assay

The antiproliferative activity of the candidate compounds was assessed against epimastigotes of the Y strain of *T. cruzi*. Parasites were cultured at 28 °C in BHT medium supplemented with 20 μg/mL hemin, 10% heat-inactivated fetal bovine serum (FBS), 100 μg/mL streptomycin, and 100 U/mL penicillin. The compounds were tested at concentrations ranging from 1 to 100 μM in cultures adjusted to a density of 1 × 10^7^ cells/mL. Control conditions were treated with DMSO at concentrations equivalent to those used with the candidate compounds. All assays, including test and control groups, were conducted in triplicate. Following a four-day incubation period, parasite viability was evaluated by counting the number of live epimastigotes using a Neubauer hemocytometer chamber under a light microscope, as previously described [61]. The half-maximal effective concentration (EC_50_) values were obtained from dose-response curves, either by fitting to a sigmoidal equation (Boltzmann model) or, where appropriate, by extrapolation from linear fitting plots [62].

#### 3.3.2. Trypomastigote Assay

To assess trypomastigote viability, parasites of the RA strain were obtained at peak parasitaemia from the peripheral blood of infected mice. The parasites (1 × 10^5^ per well) were seeded in 96-well plates containing RPMI medium supplemented with 10% FBS in a final volume of 200 μL per well. Cultures were maintained at 37 °C in a 5% CO_2_ atmosphere. Each compound was tested at a fixed concentration of 20 μM, and after 24 h of incubation, the number of motile, viable parasites was determined using a hemocytometer under a light microscope [63]. The results were expressed as the percentage of viable trypomastigotes relative to the untreated control. Benznidazole (20 μM) served as a positive control, and DMSO-treated cultures acted as solvent controls. All experimental conditions, including test compounds and controls, were conducted in triplicate to ensure reproducibility and statistical robustness.

### 3.4. Inhibitory Effect on Cruzipain (CZP) Activity Assay

The inhibitory effects of the selected compounds on cruzipain (CZP) activity were assessed following partial purification of the enzyme. CZP was isolated from *T. cruzi* extracts by ammonium sulfate precipitation, followed by affinity chromatography using cystatin-sepharose (Sigma), as previously described [64]. Enzymatic activity was measured using the synthetic substrate Bz-PFR-pNA (250 μM, Sigma) in a reaction buffer containing 6.5 μM dithiothreitol (DTT) and 50 mM Tris-HCl at pH 7.0 [65]. Reactions were carried out in the presence or absence of the test compounds at a final concentration of 100 μM.

The hydrolysis of the substrate was monitored spectrophotometrically at room temperature by measuring absorbance at 410 nm for 5 min using a Beckman Coulter™ DU530 Life Science UV-vis spectrophotometer. Enzymatic activity was quantified in picomoles of hydrolyzed substrate per minute, based on an extinction coefficient of 8800 M^−1^ cm^−1^ for p-nitroanilides. The inhibitory effect of each compound was expressed as the percentage of residual CZP activity, relative to control assays conducted in the absence of inhibitors. E64 (*N*-[*N*-(l-3-*trans*-carboxyoxirane-2-carbonyl)-l-leucyl]-agmatine), an irreversible, potent, highly selective cysteine protease inhibitor (100 μM), served as a positive control, and DMSO acted as solvent control.

### 3.5. Tryparedoxin Peroxidase (cTXNPx) and Glutathione Peroxidase I (TcGPx-I) Activity Assays

Recombinant *T. cruzi* proteins, trypanothione reductase (TcTR), tryparedoxin I (TXN-I), cytoplasmic tryparedoxin peroxidase (cTXNPx), and glutathione peroxidase I (GPx-I), were expressed in *Escherichia coli* BL21(DE3) as *N*-terminal His-tag fusions and purified by immobilized metal affinity chromatography (IMAC), following established protocols [66].

Peroxidase activity for both assays was determined spectrophotometrically by monitoring NADPH oxidation at 340 nm. The standard reaction mixture comprised 100 mM Tris-HCl (pH 7.5), 2 mM EDTA, 300 μM NADPH, 25 μM T(SH)_2_, 0.1 μM TcTR, 1 μM TcTXN-I, and 100 μM test compound. For the tryparedoxin peroxidase assay, 0.5 μM TcTXNPx was included, whereas the glutathione peroxidase I assay utilized 0.5 μM TcGPx-I. Reactions were initiated by adding 70 μM *tert*-butyl hydroperoxide (*t*-BuOOH), with two control configurations employed: a positive control omitting test compounds and a negative control excluding both test compounds and *t*-BuOOH.

Activity calculations used a molar extinction coefficient of 6.23 mM^−1^ cm^−1^ at 340 nm [66]. All enzymatic assays were conducted at 30 °C in 50 μL reaction volumes using a Multiskan Ascent photometer (Thermo Electron Co., Waltham, MA, USA) equipped with one-channel vertical light path filtration.

### 3.6. Toxicity Assay

#### 3.6.1. *In Vitro* Antiproliferative Assay

Normal human dermal fibroblast (NHDF) cell lines (PromoCell, Heidelberg, Germany) were cultured as monolayers in Dulbecco’s modified Eagle’s medium (DMEM; Gibco, Waltham, MA, USA) supplemented with penicillin/streptomycin (1% *v*/*v*) and 15% non-inactivated fetal bovine serum (Sigma, Ronkonkoma, NY, USA) at 37 °C in a humidified 5% CO_2_ atmosphere in 75 cm^2^ flasks (Nunc). All cell lines were routinely screened for mycoplasma contamination by PCR using specific primers. For the assay, cells were seeded into 96-well plates (Nunc) at 4000 cells per well and allowed to adhere for 24 h. They were then exposed for 72 h to three conditions: complete medium without test compound (negative control; 6 wells; 100% viability reference), complete medium containing 1% (*v*/*v*) Triton X-100 (positive control; 6 wells; complete lysis), or serial dilutions of the test compounds (3 wells per concentration). After 72 h, 20 µL of CellTiter 96^®^ AQueous One Solution MTS reagent (Promega, Madison, WI, USA) was added to each well containing 100 µL phenol-red-free DMEM and incubated for 1 h at 37 °C. Absorbance was recorded at 490 nm on a Synergy 4 multi-plate reader (BioTek, Santa Clara, CA, USA). Viability was expressed as the percentage of the mean absorbance of the negative control, set to 100%, and half-maximal inhibitory concentrations (EC_50_) were calculated by non-linear regression using GraphPad Prism (version 7.0). Each compound was evaluated in triplicate within a single run, and the entire protocol was performed in three independent experiments. Assays were accepted only when the negative control showed ≥90% viability and the positive control 0% viability relative to the negative control; only data fulfilling these criteria were included in the final analysis. A compound was designated cytotoxic if its EC_50_ value was <25 µM; values ≥25 µM or reported as “>25 µM” were classified as non-cytotoxic. Compounds marked NT (not tested) were excluded from the comparative analysis.

#### 3.6.2. *In Silico* Cytotoxicity Prediction

*In silico* cytotoxicity was predicted using a cytotoxicity endpoint based on molecular similarity and machine-learning models for toxicological endpoints, as described elsewhere [67,68,69]. For each compound, the SMILES representation was submitted and the cytotoxicity model was selected. The underlying model, developed on HepG2 assay data, was trained on 6156 compounds and evaluated on an external test set of 685 molecules using 10-fold cross-validation, achieving a balanced accuracy of 85% and an area under the ROC curve (AUC) of 89%. Molecular fingerprints were used as descriptors; model performance was improved by over-sampling techniques, and the applicability domain was defined by a similarity-based measure.

For the cytotoxicity endpoint, the prediction interface reports the probability of belonging to the non-cytotoxic (inactive) class, P(inactive), on a continuous scale between 0 and 1. Compounds with P(inactive) ≥0.5 are assigned to the inactive (IA, non-cytotoxic) class, whereas those with P(inactive) <0.5 are assigned to the active (AC, cytotoxic) class. In this study, this default 0.5 threshold on P(inactive) was retained, and both the categorical assignment (IA vs. AC) and the associated probability were recorded for subsequent analysis.

#### 3.6.3. Comparative Toxicity Analysis

Toxicity predictions were benchmarked against experimental NHDF EC_50_ data by constructing a 2 × 2 contingency table. Experimentally, compounds with EC_50_ < 25 µM were classified as cytotoxic (CT), whereas those with EC_50_ ≥ 25 µM or reported as “>25 µM” were classified as non-cytotoxic (NC). *In silico*, compounds with P(inactive) ≥0.5 were assigned to the inactive (IA, predicted non-cytotoxic) class and those with P(inactive) <0.5 to the active (AC, predicted cytotoxic) class. On this basis, true positives (TP) were defined as AC/CT, true negatives (TN) as IA/NC, false positives (FP) as AC/NC, and false negatives (FN) as IA/CT.

Performance metrics were then computed as follows: overall accuracy = (TP + TN)/(TP + TN + FP + FN); sensitivity = TP/(TP + FN); specificity = TN/(TN + FP); positive predictive value (PPV) = TP/(TP + FP); and negative predictive value (NPV) = TN/(TN + FN). Compounds without experimental cytotoxicity data (NT) were excluded from these calculations but were retained for qualitative structural analysis. Structural inspection of misclassified compounds (FP and FN) was undertaken to identify potential scaffold-dependent or substituent-dependent alerts that are not captured by the *in silico* cytotoxicity model.

### 3.7. Computational Methods

#### 3.7.1. Covalent Pose Modeling

The TSC scaffold is known to interact with CZP through a 1,2-polar Michael addition mechanism involving the catalytic cysteine residue (Cys26) thiolate and the thiocarbonyl group (C=S) of the TSC derivatives [29,70,71]. The nature of the reactive group and the stability of the bond formed between the enzyme and the ligand determine whether the inhibitory effect is reversible or irreversible. Additionally, the recognition group plays a crucial role, not only in ensuring affinity but also in conferring selectivity to the interaction [71]. Consequently, the tetrahedral thioacyl adduct resulting from this interaction was adopted as the initial model for covalent pose studies. Given that the thio group of the TSC scaffold forms a prochiral center, and the stereochemistry of the reactive intermediate is not known a priori, both *S*- and *R*-stereoisomers were generated and evaluated during covalent pose procedures. This mechanism of reversible covalent interaction between TSC derivatives and CZP, previously supported by experimental and computational studies [29,72], served as the basis for our covalent pose strategy. To further refine the binding pose and improve complex stability, covalent CZP-TSC MD simulations were performed, followed by binding free energy scoring using the MM-GBSA approach [73,74].

#### 3.7.2. Binding Pose Refinement

To account for solvent and thermal fluctuations in CZP-TSC binding pose and affinity, MD simulations were performed. The initial conformation of CZP was obtained from the high-resolution crystal structure deposited in the Protein Data Bank (PDB ID: 3I06) [75]. The protein was parameterized using the ff19SB force field, while parameters for the TSC ligands were derived using the GAFF2 force field. Each CZP-TSC complex was solvated in a cubic box of TIP3P water molecules, maintaining a minimum buffer of 10 Å from the protein surface. Systems were neutralized with counterions. Simulations employed periodic boundary conditions under the NPT ensemble (constant number of particles, pressure, and temperature), with temperature regulation via a Langevin thermostat. Long-range electrostatic interactions were treated using the particle-mesh Ewald method. Energy minimization, equilibration, and production runs were carried out using the AMBER suite. Initially, systems were minimized for 8000 steps using the steepest descent method. Subsequently, a 10 ns equilibration phase under the NVT ensemble (constant number of particles, volume, and temperature) was conducted, wherein ligands were positionally restrained while allowing protein relaxation. Following equilibration, restraints were removed, and production simulations were performed at 300 K and 1 atm with an integration time step of 2 fs. Production runs extended for 50 ns per complex, from which CZP-TSC interaction energies were extracted. Complex stability throughout the simulations was assessed by calculating the root mean square deviation (RMSD) of atomic positions relative to the starting structures.

#### 3.7.3. Binding Free Energy Scoring

Post-simulation binding free energy scoring was carried out using the MM/PB(GB)SA methods [73,76]. Within the MM/PB(GB)SA framework, the binding free energy (ΔG_bind_) is estimated as an average over snapshots extracted from the molecular dynamics (MD) trajectory of the protein-ligand complex, according to the expression: ΔG_bind_ = <G_PL_ − G_P_ − G_L_ > _PL_, where G_PL_, G_P_, and G_L_ denote the free energies of the protein-ligand complex, the unbound protein, and the unbound ligand, respectively. Each free energy term G is computed as: G = E_MM_ + E_vdW_ + G_pol_ + G_np_ − TS. Here, E_MM_ corresponds to the molecular mechanics energy (including bond, angle, and dihedral terms), electrostatic, and van der Waals interactions. G_pol_ and G_np_ represent the polar and nonpolar components of solvation free energy, respectively. The polar contribution is typically calculated either by solving the Poisson-Boltzmann (PB) equation or by applying the Generalized Born (GB) approximation, whereas the non-polar contribution is estimated from a linear relationship with the solvent-accessible surface area (SASA). Finally, the entropic contribution (TS) is obtained through normal-mode analysis of vibrational frequencies at a given absolute temperature (T) [73,74].

#### 3.7.4. Tanimoto Similarity Analysis

A comparative analysis of structural similarity was carried out for the twenty TSC derivatives, using SMILES notations. The molecular descriptors were calculated from SMILES strings and computed with in-house Python code (version 3.9) under fixed algorithmic parameters for reproducibility. After the conversion of SMILES representations to molecular graphs, the structural consistency was checked with the cheminformatics toolkit RDKit (version 2022.09.5). The Morgan algorithms with radius 2 were employed to create 1024-bit molecular fingerprints recording substructural patterns [77], which allow for quantitative pairwise analyses.

Hierarchical cluster analysis was carried out on the Tanimoto similarity coefficients derived from the provided fingerprints. Pairwise evaluation of structural similarities between the molecules generated a symmetric 20 × 20 similarity matrix [78,79] with compound identifier annotations and numeric values rounded to three decimal places. To visualize these relationships, a dual-tone heatmap with a blue-to-red color scale was constructed. Dendrograms were generated using average linkage clustering based on Tanimoto distances calculated as 1 minus the similarity. This method arranges structurally similar compounds along both axes, making it easier to visually interpret patterns of structural connectivity and similarity within the dataset.

### 3.8. Statistical Analysis

Unless otherwise stated, biological assay results are presented as mean ± SD from replicate determinations performed as described in Section 3.2, Section 3.3, Section 3.4, Section 3.5 and Section 3.6. Epimastigote EC_50_ values were obtained from dose-response curves generated using serial dilutions and quantified by hemocytometer counting, following the procedure described in Section 3.2. Trypomastigote viability (Section 3.3) and enzymatic residual activities (Section 3.4, Section 3.5 and Section 3.6) are reported as percentages relative to the assay-specific controls defined for each experiment. NHDF cytotoxicity values are reported as mean ± SEM from three independent measurements (Section 3.6.3). Because the study design was intended as a primary screening workflow and was not based on a pre-specified hypothesis-testing plan across multiple endpoints, *p*-values are not reported.

## 4. Conclusions

This study investigated the synthesis and antitrypanosomal profiling of a new series of piperazinyl/morpholinyl thiosemicarbazone (TSC) derivatives, with emphasis on how substituent patterns, steric effects, molecular conformation, and binding geometry relate to stage-specific phenotypic responses. Within the series, derivative **3e**, bearing nitro and methoxy substituents, emerged as the most potent cruzipain (CZP) inhibitor. Its activity was consistent with a favorable *S*-pose binding mode and produced near-complete loss of epimastigote viability. However, **3e** showed limited efficacy against bloodstream trypomastigotes, indicating that CZP-centered activity does not necessarily translate to the infective stage under the screening conditions and supporting the likelihood that additional, CZP-independent mechanisms contribute to activity in this life stage.

In contrast, the brominated analogues **2c** and **2e** displayed dual-stage efficacy against both epimastigotes and trypomastigotes despite weak or unclear CZP inhibition, suggesting engagement of other enzymatic or cellular targets. Structure-activity relationships supported by Tanimoto similarity clustering associated nitro-methoxy substitution with epimastigote-selective profiles and brominated scaffolds with broader antiparasitic effects. Outliers such as **7b** and **10e**, together with the practical constraints observed across the set, further highlight that an appropriate balance between hydrophobicity and steric complementarity is required for biological activity. The absence of measurable inhibition of the redox enzymes cytosolic tryparedoxin peroxidase (cTXNPx) and glutathione peroxidase type I (TcGPx-I), along with reduced performance of bulkier substituents, is consistent with stage-specific TSC activity involving more than one mode of action.

Overall, cytotoxicity data indicate that derivatives **2a–10e** should be viewed as starting points for optimization rather than development-ready candidates, as most compounds showed low or no non-specific toxicity toward fibroblasts under the tested conditions, but safety remains scaffold-dependent. In this context, combining epimastigote and trypomastigote phenotypic assays with enzyme inhibition and cytotoxicity testing provides a rational first-pass filter to prioritize scaffolds with direct trypanocidal activity and potential to limit parasite spread, while reserving intracellular amastigote models for downstream validation. Because trypomastigote response was assessed at a single concentration and intracellular amastigote assays were not included in the present design, further studies will be required to establish exposure-response relationships and confirm translational relevance in intracellular infection settings.

## Figures and Tables

**Table 1:** Trypanocidal activity in epimastigote and trypomastigote assays, enzymatic inhibition assays, and covalent docking results for the tested compounds. Epimastigote potency is reported as EC_50_ (µM). Trypomastigote viability is reported at 20 µM as percent viability relative to the negative control. Enzyme results are reported at 100 µM as residual activity (percent) relative to assay-specific controls. Covalent docking results are reported as binding free energies (ΔG, kcal/mol) for tetrahedral thioacyl adduct poses (*R* and *S*).

Comp.	Epimastigote EC_50_ (µM) ^1^	Trypomastigote Viability at 20 µM (%) ^2^	CZP Residual Activity at 100 µM (%) ^3^	cTXNPx Residual Activity at 100 µM (%) ^3^	TcGPx-I Residual Activity at 100 µM (%) ^3^	ΔG R-Pose (kcal/mol) ^4^	ΔG S-Pose (kcal/mol) ^4^
**2a**	4.11 ± 0.45	97.35 ± 0.90	77.15 ± 2.95	93.93 ± 2.85	75.92 ± 3.96	−17.02 ± 0.99	−16.54 ± 1.09
**2b**	27.63 ± 4.63	99.70 ± 1.27	83.27 ± 1.90	71.60 ± 4.86	68.19 ± 6.03	−18.51 ± 0.89	−18.79 ± 1.27
**2c**	3.92 ± 0.10	0.00 ± 0.00	0.00 ± 0.00	76.07 ± 2.37	65.77 ± 6.08	−15.78 ± 1.00	−15.95 ± 1.04
**2d**	5.88 ± 0.74	99.50 ± 1.49	0.00 ± 0.00	85.18 ± 1.73	67.90 ± 5.84	−17.10 ± 0.96	−18.85 ± 0.86
**2e**	4.36 ± 0.69	0.00 ± 0.00	0.00 ± 0.00	78.82 ± 1.74	67.94 ± 4.45	−15.79 ± 0.91	−15.75 ± 1.11
**2f**	10.85 ± 1.71	100.36 ± 1.82	0.00 ± 0.00	80.29 ± 3.26	69.96 ± 5.88	−15.08 ± 0.89	−14.95 ± 0.73
**2g**	13.38 ± 5.51	99.65 ± 2.26	0.00 ± 0.00	75.47 ± 1.19	68.88 ± 4.36	−15.64 ± 0.92	−14.15 ± 0.78
**3b**	4.19 ± 0.45	0.00 ± 0.00	89.01 ± 7.70	90.99 ± 1.55	71.74 ± 5.47	−19.31 ± 4.03	−24.72 ± 3.52
**3e**	0.36 ± 0.07	80.29 ± 2.76	0.00 ± 0.00	63.79 ± 2.70	72.38 ± 6.23	−19.33 ± 0.93	−20.78 ± 1.85
**3f**	12.76 ± 3.35	101.85 ± 2.92	0.00 ± 0.00	70.52 ± 3.31	69.41 ± 6.46	−16.44 ± 0.83	−15.83 ± 0.95
**3g**	20.55 ± 5.62	97.50 ± 1.95	88.01 ± 6.45	76.84 ± 2.80	71.11 ± 5.52	−18.89 ± 0.82	−19.73 ± 0.93
**4b**	18.32 ± 2.15	100.00 ± 0.00	69.95 ± 4.20	75.00 ± 3.06	61.83 ± 4.40	−16.41 ± 1.00	−15.52 ± 1.22
**4c**	11.35 ± 1.54	0.00 ± 0.00	0.00 ± 0.00	76.04 ± 2.37	72.99 ± 6.48	−17.00 ± 0.93	−16.26 ± 0.92
**4e**	4.84 ± 2.05	97.30 ± 2.90	95.34 ± 4.73	75.00 ± 1.73	60.33 ± 5.21	−17.44 ± 0.86	−17.37 ± 0.83
**5a**	4.89 ± 0.75	100.10 ± 1.41	83.67 ± 5.16	90.18 ± 3.00	69.52 ± 4.25	−17.47 ± 0.96	−17.11 ± 0.98
**6c**	9.06 ± 2.02	100.00 ± 0.00	84.82 ± 1.85	77.75 ± 2.18	64.66 ± 5.85	−20.06 ± 3.34	−22.59 ± 3.52
**7b**	13.33 ± 0.40	1.50 ± 2.12	0.00 ± 0.00	83.42 ± 2.20	75.07 ± 6.71	−16.47 ± 0.78	−15.75 ± 0.86
**8a**	15.39 ± 2.66	97.90 ± 0.14	85.12 ± 6.30	80.66 ± 2.95	60.50 ± 5.81	−16.98 ± 0.96	−17.54 ± 0.95
**9g**	2.30 ± 0.52	63.20 ± 3.40	0.00 ± 0.00	67.53 ± 2.60	66.84 ± 5.29	−18.91 ± 0.80	−19.57 ± 0.90
**10e**	10.48 ± 3.07	100.20 ± 1.69	0.00 ± 0.00	92.51 ± 1.94	70.81 ± 5.36	−17.95 ± 0.88	−17.45 ± 0.84
**Ctrl (-)**	—	100.00 ± 6.47	100.00 ± 1.97	2.00 ± 0.06	1.00 ± 0.03	—	—
**Ctrl (+)**	—	4.75 ± 0.33	0.00 ± 0.00	100.00 ± 1.72	100.00 ± 3.75	—	—

^1^ Epimastigote EC_50_ (µM) after 4 days of exposure. ^2^ Trypomastigote viability (%) after 24 h exposure at 20 µM, reported relative to the negative control (100%). ^3^ Residual enzymatic activity (%) at 100 µM. For CZP, the negative control corresponds to the assay without inhibitor (100%) and the positive control corresponds to the reference inhibitor (0%). For cTXNPx and TcGPx-I, the positive control corresponds to the complete reaction without test compound (100%), while the negative control corresponds to omission of t-BuOOH (near-baseline activity). ^4^ ΔG values (kcal/mol) from covalent docking for *R*- and *S*-pose tetrahedral thioacyl adducts.

**Table 2:** Experimental cytotoxicity CC_50_ values in NHDF compared with *in silico* prediction and epimastigote selectivity indices.

Comp.	NHDF CC_50_ (µM) ± SEM ^1^	Calculated P(inactive)	Calculated Class ^2^	Experimental Class ^3^	Concordance ^4^	Epimastigote SI (CC_50_/EC_50_) ^5^
**2a**	>25	0.59	IA	NC	TN	>6.08
**2b**	NT	0.62	IA	NT	NT	NT
**2c**	14.4 ± 0.7	0.61	IA	CT	FN	3.67
**2d**	21.5 ± 3.0	0.61	IA	CT	FN	3.66
**2e**	17.0 ± 3.7	0.57	IA	CT	FN	3.90
**2f**	NT	0.61	IA	NT	NT	NT
**2g**	>25	0.61	IA	NC	TN	>1.87
**3b**	>25	0.66	IA	NC	TN	>5.97
**3e**	>25	0.66	IA	NC	TN	>69.44
**3f**	>25	0.65	IA	NC	TN	>1.96
**3g**	>25	0.64	IA	NC	TN	>1.22
**4b**	>25	0.66	IA	NC	TN	>1.37
**4c**	>25	0.70	IA	NC	TN	>2.20
**4e**	>25	0.66	IA	NC	TN	>5.17
**5a**	>25	0.62	IA	NC	TN	>5.11
**6c**	NT	0.61	IA	NT	NT	NT
**7b**	>25	0.62	IA	NC	TN	>1.88
**8a**	>25	0.56	IA	NC	TN	>1.62
**9g**	>25	0.62	IA	NC	TN	>10.87
**10e**	NT	0.59	IA	NT	NT	NT

^1^ CC_50_ values were determined by treating NHDF with different concentrations of each compound and calculating EC_50_/CC_50_ values from three independent measurements; results are presented as mean ± SEM. NT = not tested. “>25 µM” indicates no significant cytotoxicity up to the maximum concentration tested. ^2^ *In silico* classification threshold: P(inactive) ≥0.5 = inactive (IA); P(inactive) <0.5 = active (AC). All predictions were generated using the cytotoxicity model. ^3^ Experimental class based on EC_50_: Cytotoxic (CT) = EC_50_ <25 µM; Non-cytotoxic (NC) = EC_50_ ≥25 µM. ^4^ Concordance categories: TN = true negative; FN = false negative; NT = not testable. ^5^ Selectivity index (SI) calculated as CC_50_/epimastigote EC_50_ using Table 1 EC_50_ values; for entries reported as “>25 µM”, SI is reported as a lower bound.

## Data Availability

The original contributions presented in this study are included in the article/Supplementary Materials. Further inquiries can be directed to the corresponding authors.

## Supplementary Materials

The following supporting information can be downloaded at: https://www.mdpi.com/article/10.3390/ph19010182/s1, Figure S1: ^1^H-NMR (DMSO-*d_6_*) spectrum of *N*′-[(5-bromo-2-hydroxyphenyl)methylidene]-4-(4-methoxyphenyl)piperazine-1-carbothiohydrazide (**2b**); Figure S2: ^13^C-NMR (DMSO-*d_6_*) spectrum of *N*′-[(5-bromo-2-hydroxyphenyl)methylidene]-4-(4-methoxyphenyl)piperazine-1-carbothiohydrazide (**2b**); Figure S3: ^1^H-NMR (DMSO-*d_6_*) spectrum of *N*′-[(5-bromo-2-hydroxyphenyl)methylidene]-4-(4-fluorophenyl)piperazine-1-carbothiohydrazide (**2c**); Figure S4: ^13^C-NMR (DMSO-*d_6_*) spectrum of *N*′-[(5-bromo-2-hydroxyphenyl)methylidene]-4-(4-fluorophenyl)piperazine-1-carbothiohydrazide (**2c**); Figure S5: ^1^H-NMR (DMSO-*d_6_*) spectrum of *N*′-[(5-bromo-2-hydroxyphenyl)methylidene]-4-(4-nitrophenyl)piperazine-1-carbothiohydrazide (**2e**); Figure S6: ^13^C-NMR (DMSO-*d_6_*) spectrum of *N*′-[(5-bromo-2-hydroxyphenyl)methylidene]-4-(4-nitrophenyl)piperazine-1-carbothiohydrazide (**2e**); Figure S7: ^1^H-NMR (DMSO-*d_6_*) spectrum of *N*′-[(5-bromo-2-hydroxyphenyl)methylidene]-4-phenylpiperazine-1-carbothiohydrazide (**2f**); Figure S8: ^13^C-NMR (DMSO-*d_6_*) spectrum of *N*′-[(5-bromo-2-hydroxyphenyl)methylidene]-4-phenylpiperazine-1-carbothiohydrazide (**2f**); Figure S9: ^1^H-NMR (DMSO-*d_6_*) spectrum of *N*′-[(4-hydroxy-3-methoxyphenyl)methylidene]-4-(4-methoxyphenyl)piperazine-1-carbothiohydrazide (**3b**); Figure S10: ^13^C-NMR (DMSO-*d_6_*) spectrum of *N*′-[(4-hydroxy-3-methoxyphenyl)methylidene]-4-(4-methoxyphenyl)piperazine-1-carbothiohydrazide (**3b**); Figure S11: ^1^H-NMR (DMSO-*d_6_*) spectrum of *N*′-[(4-hydroxy-3-methoxyphenyl)methylidene]-4-(4-nitrophenyl)piperazine-1-carbothiohydrazide (**3e**); Figure S12: ^13^C-NMR (DMSO-*d_6_*) spectrum of *N*′-[(4-hydroxy-3-methoxyphenyl)methylidene]-4-(4-nitrophenyl)piperazine-1-carbothiohydrazide (**3e**); Figure S13: ^1^H-NMR (DMSO-*d_6_*) spectrum of *N*′-[(4-hydroxy-3-methoxyphenyl)methylidene]morpholine-4-carbothiohydrazide (**3g**); Figure S14: ^13^C-NMR (DMSO-*d_6_*) spectrum of *N*′-[(4-hydroxy-3-methoxyphenyl)methylidene]morpholine-4-carbothiohydrazide (**3g**); Figure S15: ^1^H-NMR (DMSO-*d_6_*) spectrum of *N*′-[(2-fluorophenyl)methylidene]-4-(4-methoxyphenyl)piperazine-1-carbothiohydrazide (**4b**); Figure S16: ^13^C-NMR (DMSO-*d_6_*) spectrum of *N*′-[(2-fluorophenyl)methylidene]-4-(4-methoxyphenyl)piperazine-1-carbothiohydrazide (**4b**); Figure S17: ^1^H-NMR (DMSO-*d_6_*) spectrum of *N*′-[(2-fluorophenyl)methylidene]-4-(4-fluorophenyl)piperazine-1-carbothiohydrazide (**4c**); Figure S18: ^13^C-NMR (DMSO-*d_6_*) spectrum of *N*′-[(2-fluorophenyl)methylidene]-4-(4-fluorophenyl)piperazine-1-carbothiohydrazide (**4c**); Figure S19: ^1^H-NMR (DMSO-*d_6_*) spectrum of ***N*′**-[(2-fluorophenyl)methylidene]-4-(4-nitrophenyl)piperazine-1-carbothiohydrazide (**4e**); Figure S20: ^13^C-NMR (DMSO-*d_6_*) spectrum of ***N*′**-[(2-fluorophenyl)methylidene]-4-(4-nitrophenyl)piperazine-1-carbothiohydrazide (**4e**); Figure S21: ^1^H-NMR (DMSO-*d_6_*) spectrum of *N*′-2{[(3,4,5-trihydroxy-6-hydroxymethylene)tetrahydropyran-2-yloxy]benzylidene}morpholine-4-carbothiohydrazide (**9g**); Figure S22: ^13^C-NMR (DMSO-*d_6_*) spectrum of *N*′-2{[(3,4,5-trihydroxy-6-hydroxymethylene)tetrahydropyran-2-yloxy]benzylidene}morpholine-4-carbothiohydrazide (**9g**); Figure S23: Interaction diagrams for compound **3e**; Figure S24: Heatmap benchmark of *in silico* predictions against experimental NHDF cytotoxicity; Table S1: Tanimoto similarity matrix between each pair of piperazinyl thiosemicarbazone derivatives **2**–**10**. The binary Extended Connectivity Fingerprints (radius = 2, bit = 1024) was used for the similarity calculation.

## Abbreviations

The following abbreviations are used in this manuscript:

AUC	Area under the ROC curve
Bz-PFR-pNA	N-benzoyl-Pro-Phe-Arg-p-nitroanilide hydrochloride
CD	Chagas disease
cTXNPx	Cytoplasmic tryparedoxin peroxidase
CZP	Cruzipain
CZP-TSC	Cruzipain-thiosemicarbazone adduct
DMSO	Dimethyl sulfoxide
DTT	Dithiothreitol
E64 inhibitor	N-[n-(l-3-trans-carboxyoxirane-2-carbonyl)-l-leucyl]-agmatine
EC_50_	Concentration of the compounded needed to induce a 50% response
FBS	Fetal bovine serum
GAFF2	General Amber force field, second generation
GSH	Glutathione
IMAC	Immobilized metal affinity chromatography
MACCS	Molecular access system fingerprint
MD	Molecular dynamics simulation
MM/PB(GB)SA	Molecular mechanics with Poisson-Boltzmann (PB) or Generalized Born (GB) surface area solvation
MM-GBSA	Molecular mechanics with Generalized Born and surface area solvation
MS	Mass spectrometry
NADPH	Reduced nicotinamide adenine dinucleotide phosphate
NHDF	Normal human dermal fibroblasts
NMR	Nuclear magnetic resonance
NPT	Constant number of particles, pressure, and temperature
NVT	Constant number of particles, volume, and temperature
RMSD	Root mean square deviation
ROC	Receiver Operating Characteristic
SAR	Structure-activity relationships
SASA	Solvent-accessible surface area
SMILES	Simplified molecular input line entry system
*T. cruzi*	*Trypanosoma cruzi*
Ta	Tanimoto similarity analysis
*t*-BuOOH	*Tert*-butyl hydroperoxide
TcGPx-I	*T. cruzi* glutathione peroxidase type I
TcTR	*T. cruzi* trypanothione reductase
TSC	Thiosemicarbazone
TXN	Tryparedoxin
TXN-I	Tryparedoxin 1
ΔG_binding_	Binding free energy
ΔG*_R_*	*R*-pose binding free energy
ΔG*_S_*	*S*-pose binding free energy
