# Supplementary material for: Integrated In Vitro and In Silico Profiling of Piperazinyl Thiosemicarbazone Derivatives Against Trypanosoma cruzi: Stage-Specific Activity and Enzyme Inhibition
Source: Pharmaceuticals (Basel). 2026 Jan 20;19(1):182. doi: 10.3390/ph19010182 (PMC12845152; doi:10.3390/ph19010182)
Supplement: Supplementary file 1 [file pharmaceuticals-19-00182-s001.zip › pharmaceuticals-4089873-supplementary.pdf]

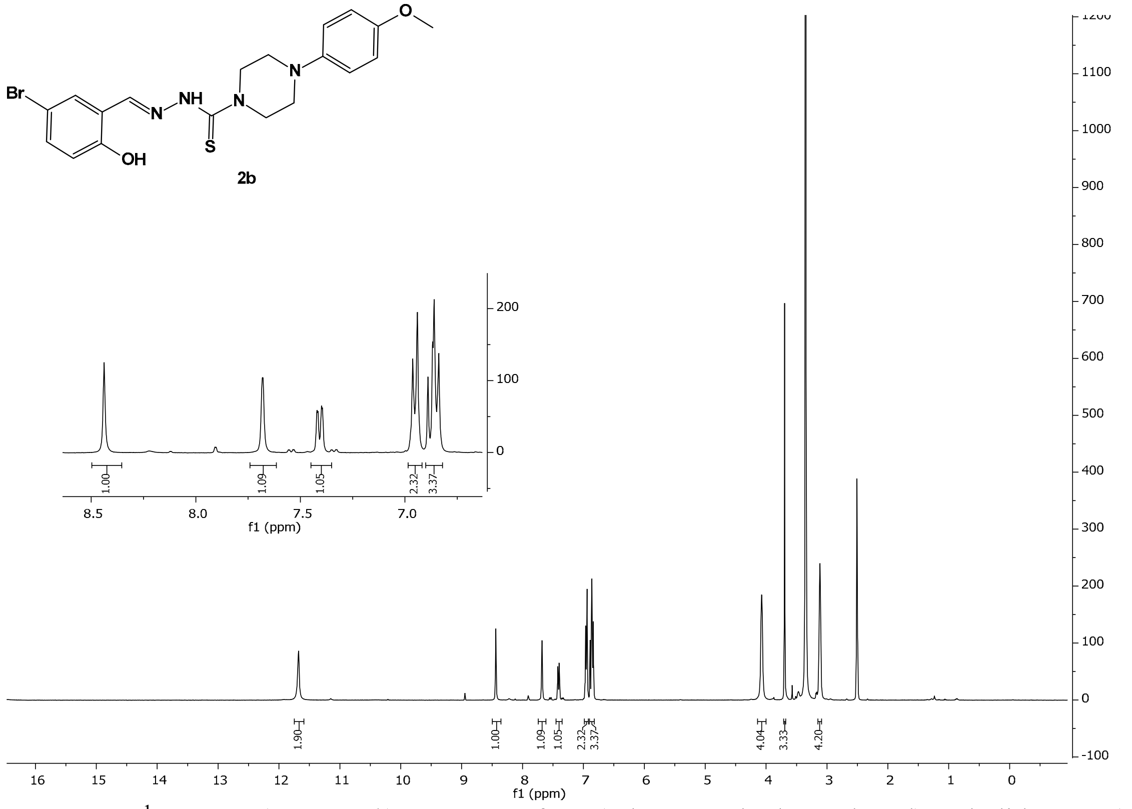

Figure S1. ^{1}H-NMR (DMSO-d_6) spectrum of *N'*-[(5-bromo-2-hydroxyphenyl)methylidene]-4-(4-methoxyphenyl)piperazine-1-carbothiohydrazide (**2b**).

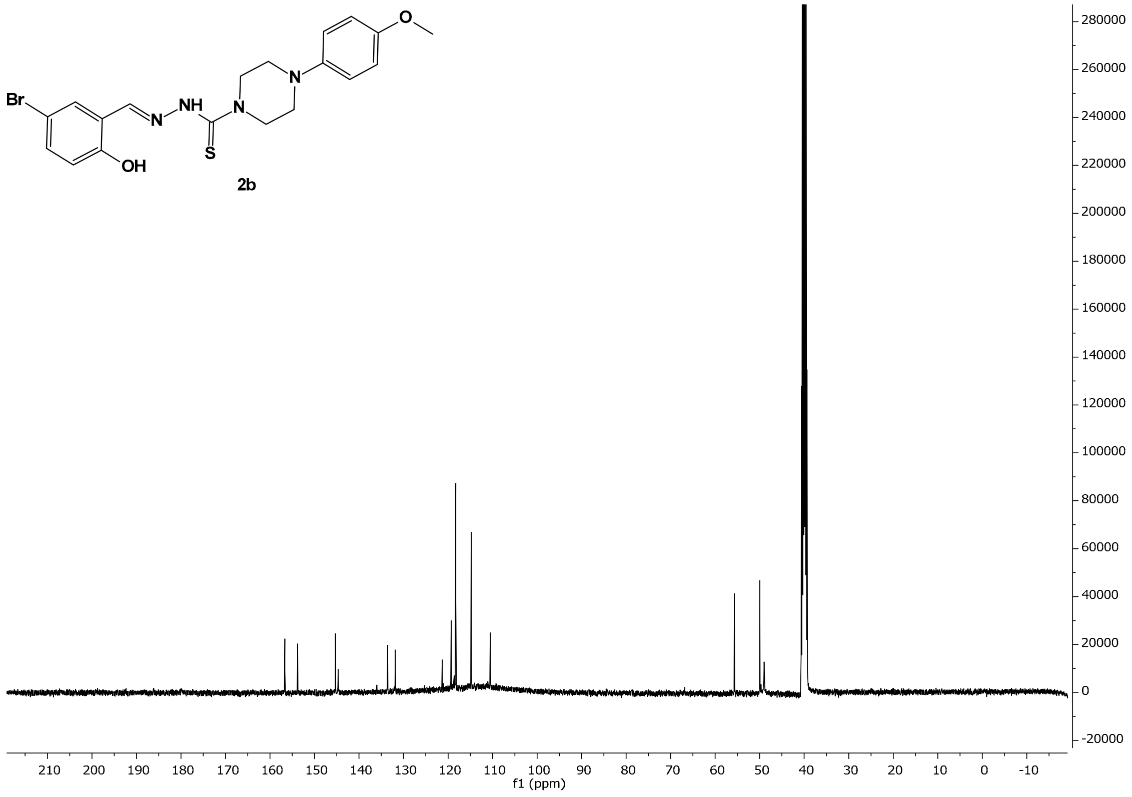

Figure S2. ^{13}C-NMR (DMSO-*d$_6$*) spectrum of *N'*-[(5-bromo-2-hydroxyphenyl)methylidene]-4-(4-methoxyphenyl)piperazine-1-carbothiohydrazide (**2b**).

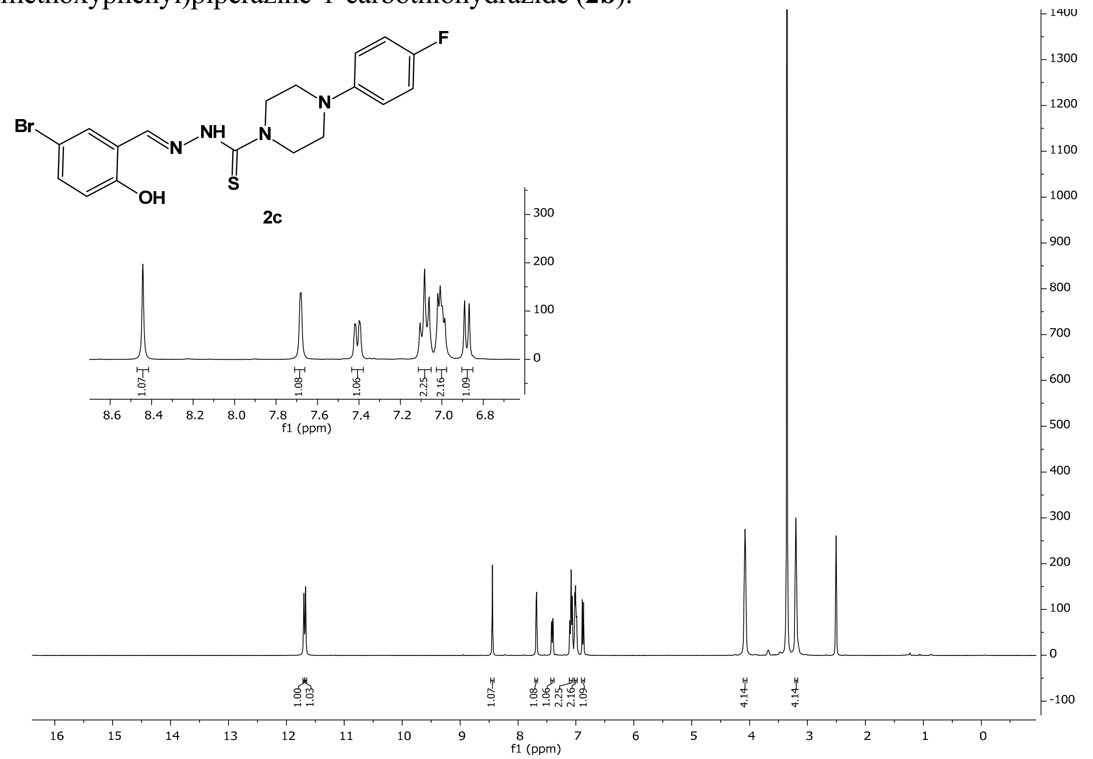

Figure S3. ^{1}H-NMR (DMSO-*d$_6$*) spectrum of *N'*-[(5-bromo-2-hydroxyphenyl)methylidene]-4-(4-fluorophenyl)piperazine-1-carbothiohydrazide (**2c**).

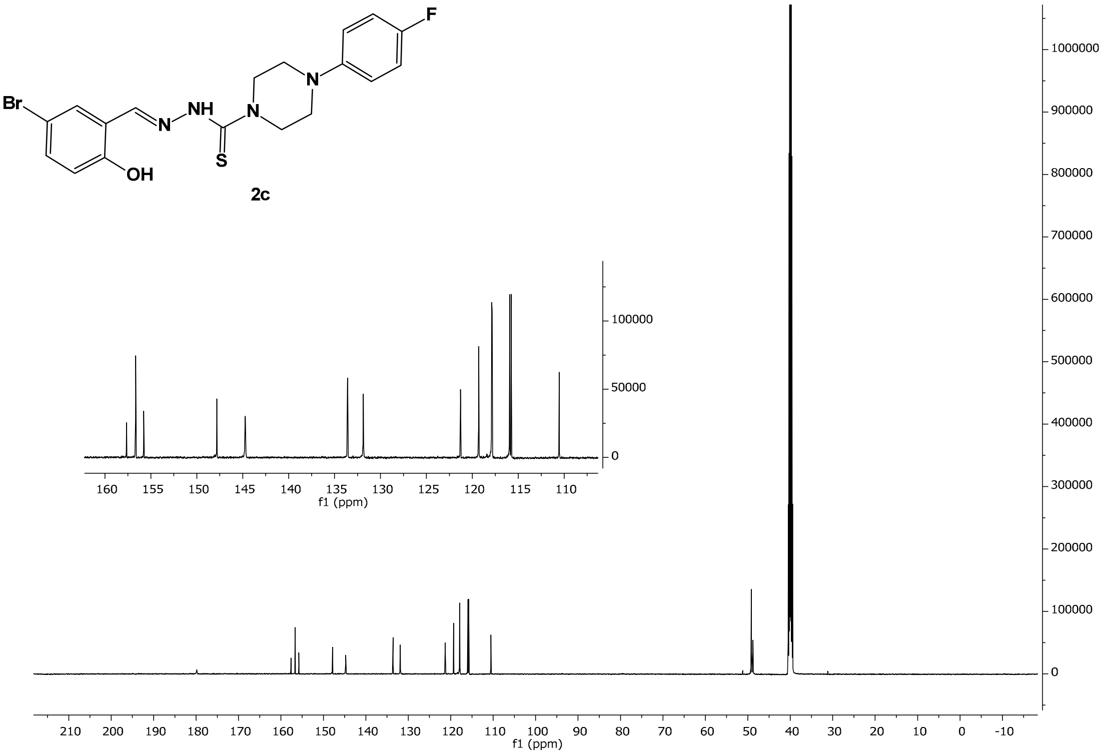

Figure S4. ^{13}C-NMR (DMSO-*d$_6$*) spectrum of *N'*-[(5-bromo-2-hydroxyphenyl)methylidene]-4-(4-fluorophenyl)piperazine-1-carbothiohydrazide (**2c**).

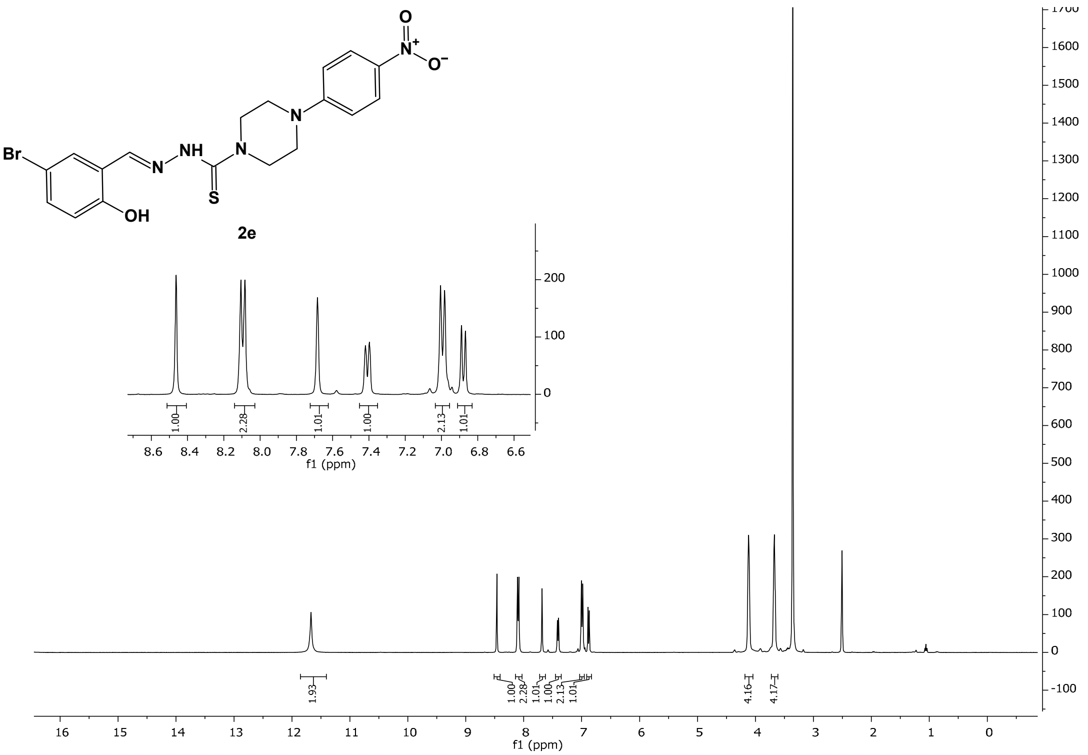

Figure S5. ^{1}H-NMR (DMSO-d_6) spectrum of *N'*-[(5-bromo-2-hydroxyphenyl)methylidene]-4-(4-nitrophenyl)piperazine-1-carbothiohydrazide (**2e**).

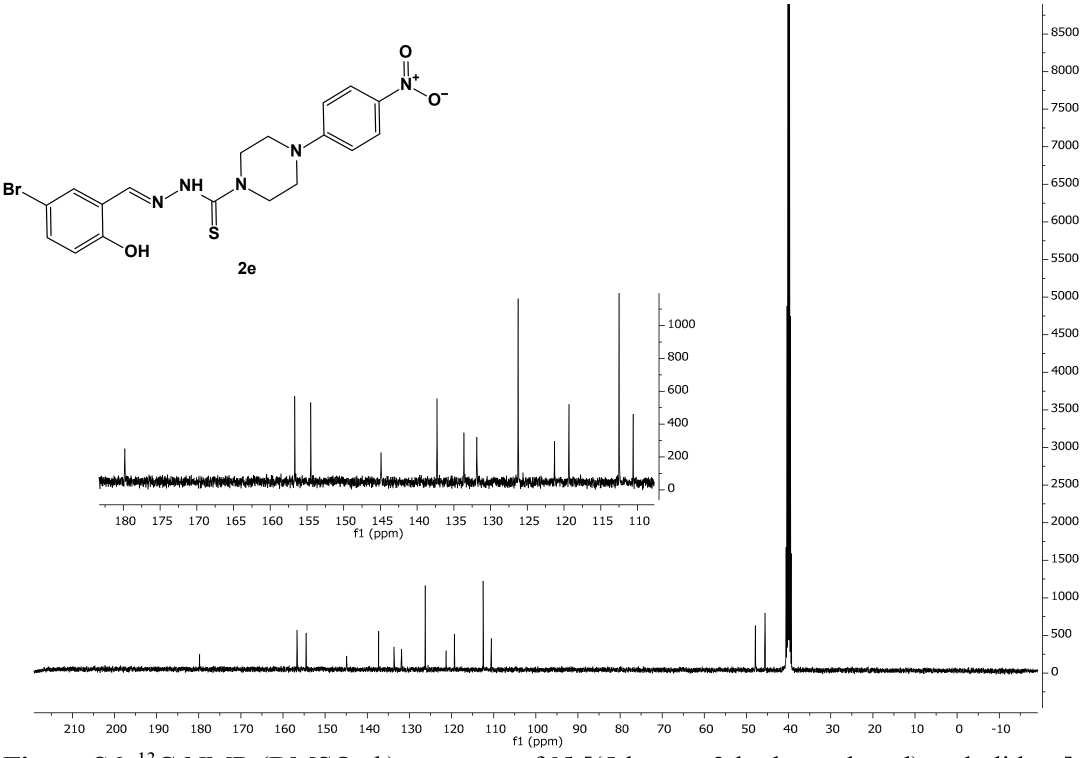

Figure S6. ^{13}C-NMR (DMSO-d_6) spectrum of *N'*-[(5-bromo-2-hydroxyphenyl)methylidene]-4-(4-nitrophenyl)piperazine-1-carbothiohydrazide (**2e**).

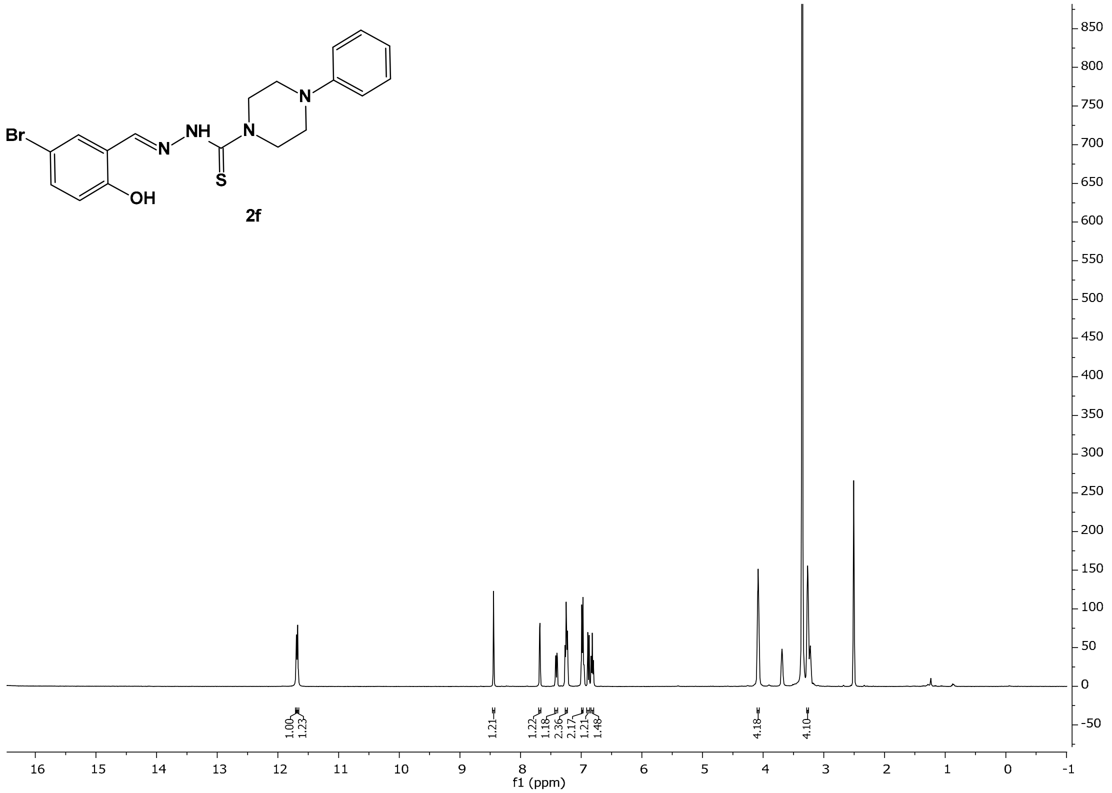

Figure S7. ^{1}H-NMR (DMSO-d_6) spectrum of *N'*-[(5-bromo-2-hydroxyphenyl)methylidene]-4-phenylpiperazine-1-carbothiohydrazide (**2f**).

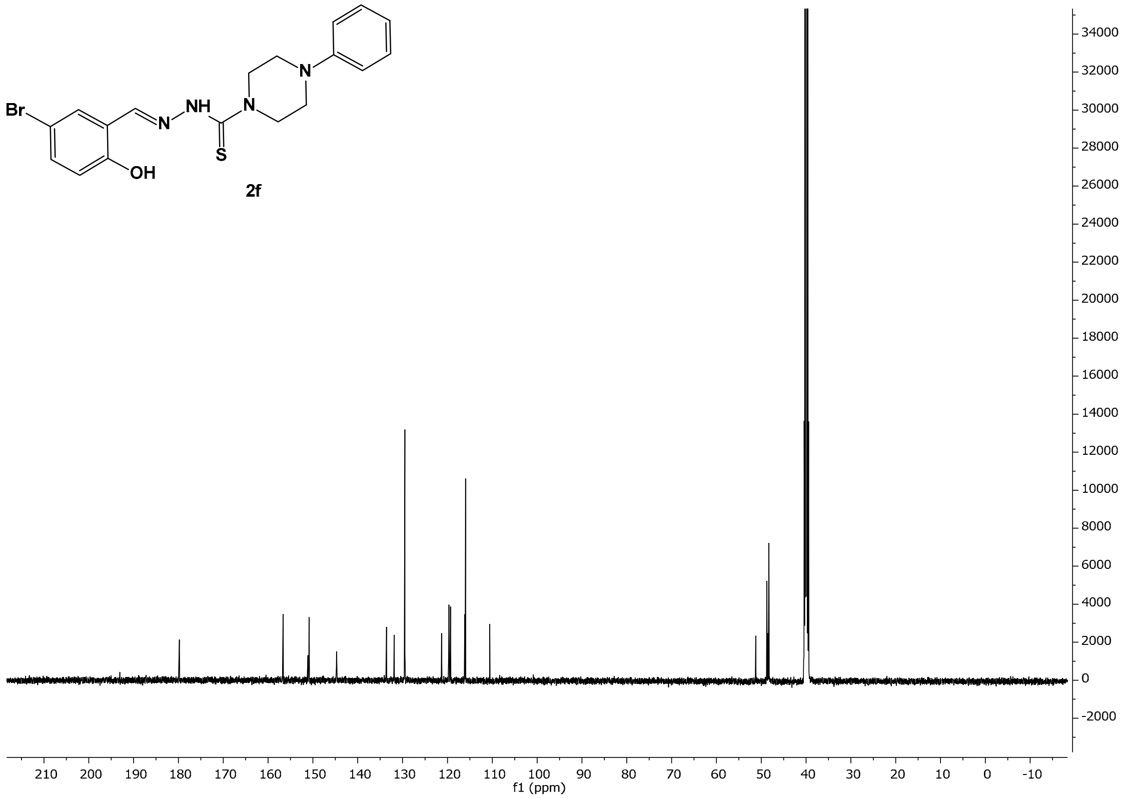

Figure S8. ^{13}C-NMR (DMSO-d_6) spectrum of *N'*-[(5-bromo-2-hydroxyphenyl)methylidene]-4-phenylpiperazine-1-carbothiohydrazide (**2f**).

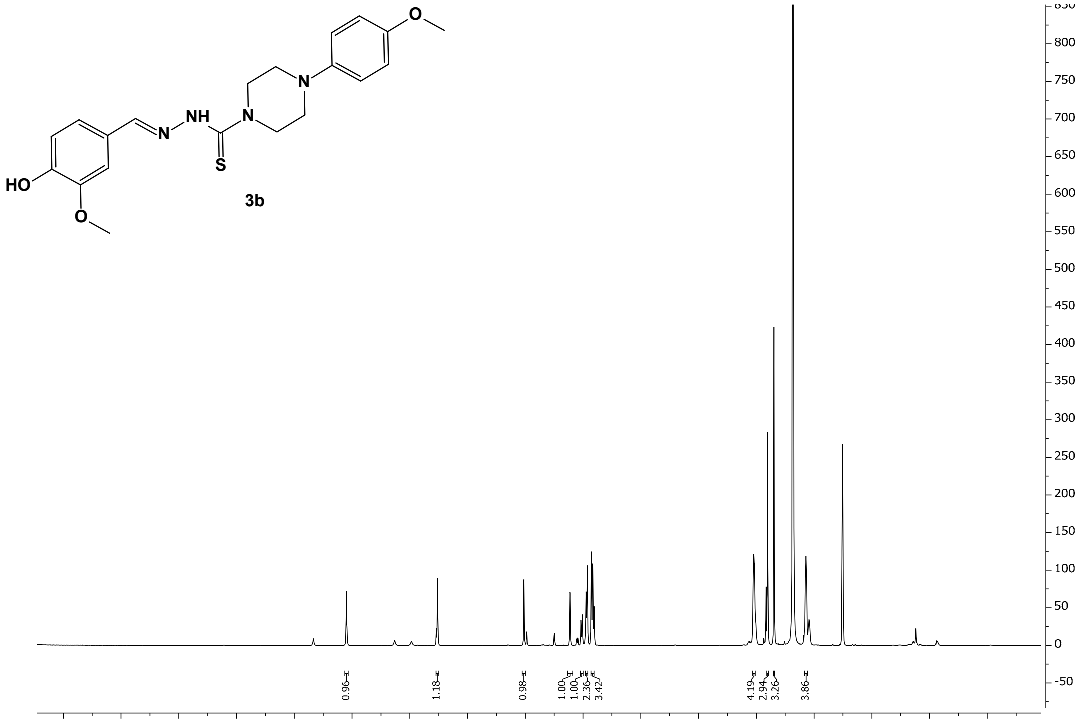

Figure S9. ^{1}H-NMR (DMSO-d_6) spectrum of *N'*-[(4-hydroxy-3-methoxyphenyl)methylidene]-4-(4-methoxyphenyl)piperazine-1-carbothiohydrazide (**3b**).

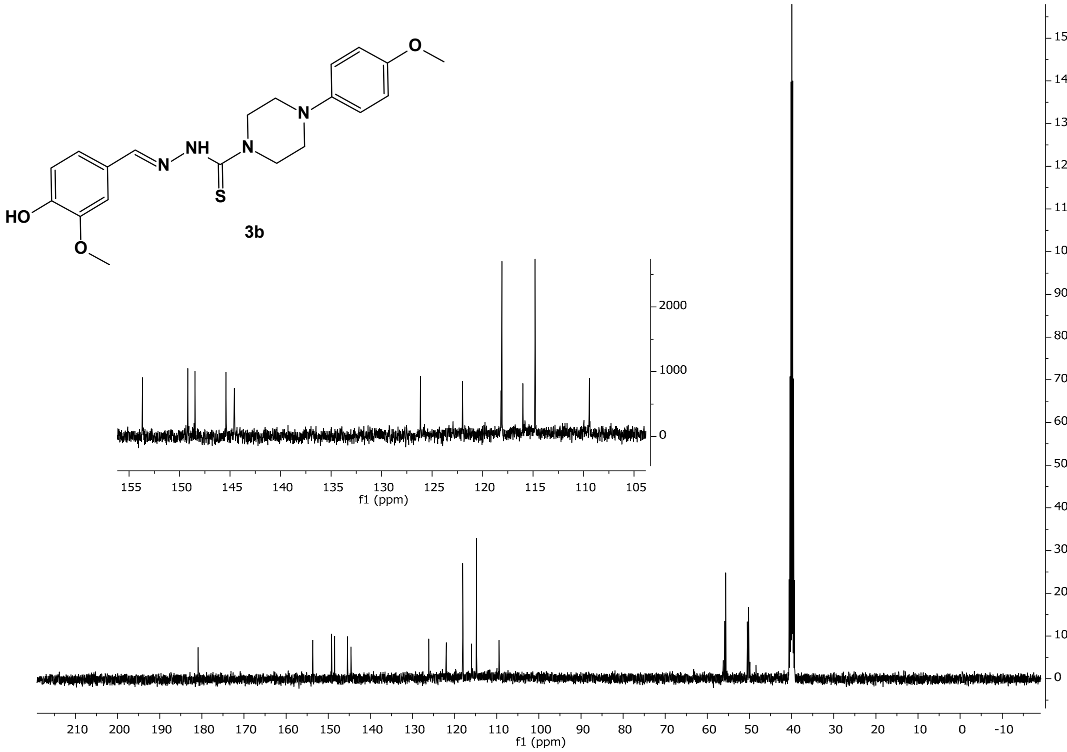

Figure S10. ^{13}C-NMR (DMSO-d_6) spectrum of *N'*-[(4-hydroxy-3-methoxyphenyl)methylidene]-4-(4-methoxyphenyl)piperazine-1-carbothiohydrazide (**3b**).

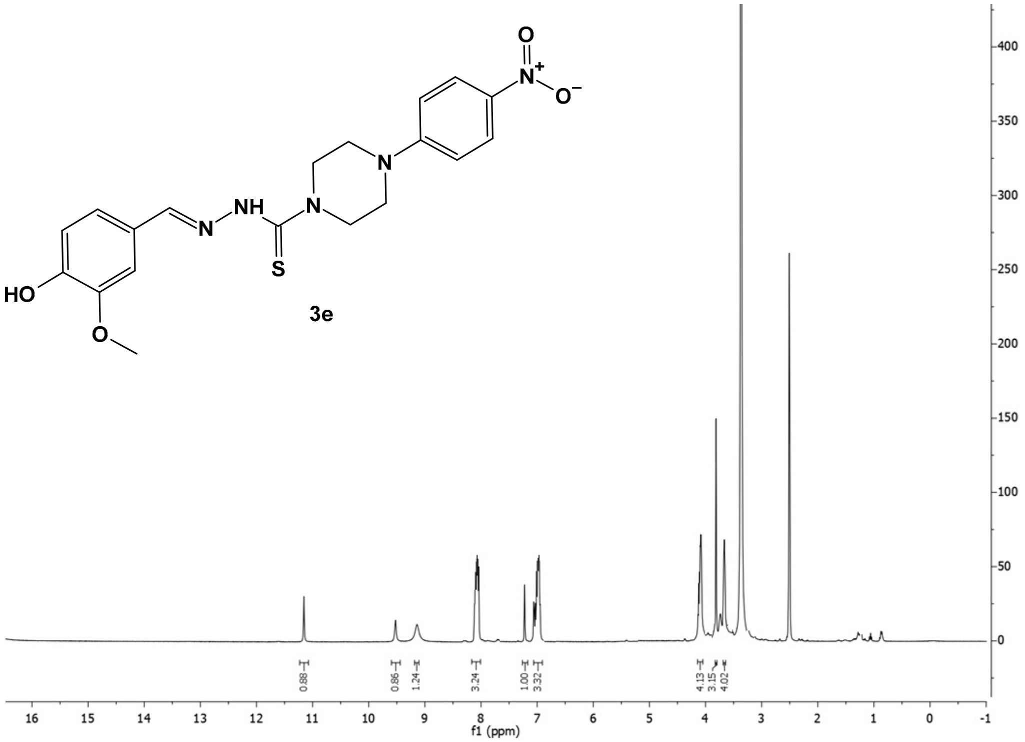

Figure S11. ^{1}H-NMR (DMSO-d_6) spectrum of *N'*-[(4-hydroxy-3-methoxyphenyl)methylidene]-4-(4-nitrophenyl)piperazine-1-carbothiohydrazide (**3e**).

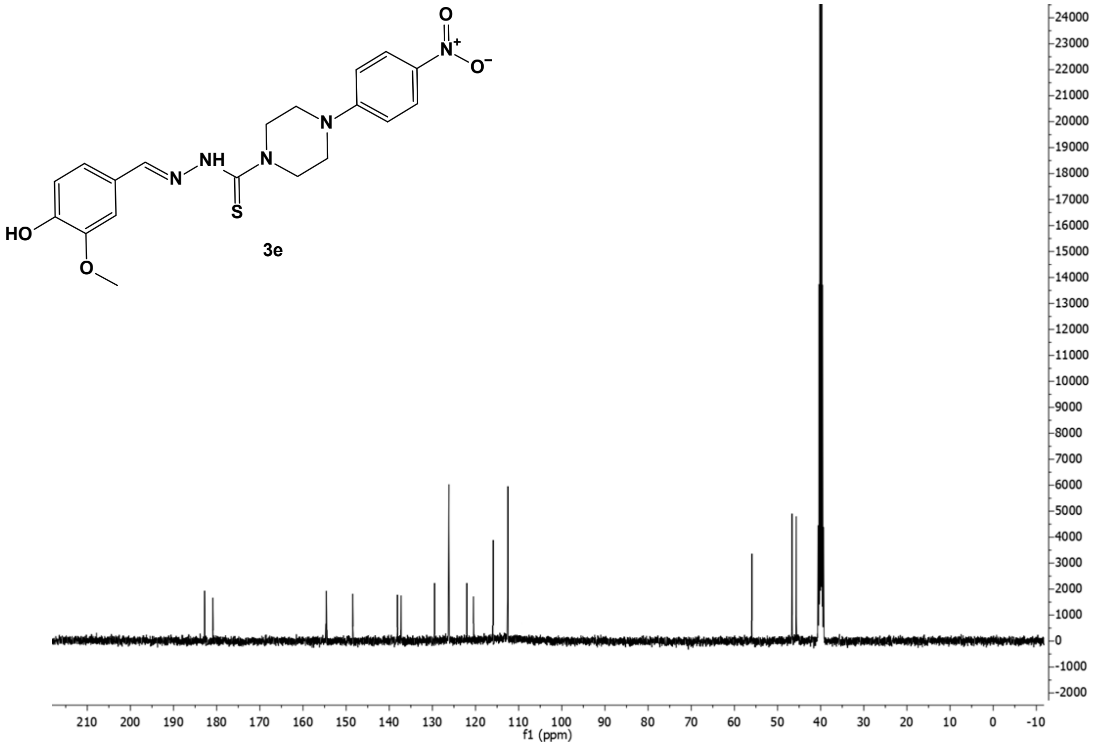

Figure S12. ^{13}C-NMR (DMSO-d_6) spectrum of *N'*-[(4-hydroxy-3-methoxyphenyl)methylidene]-4-(4-nitrophenyl)piperazine-1-carbothiohydrazide (**3e**).

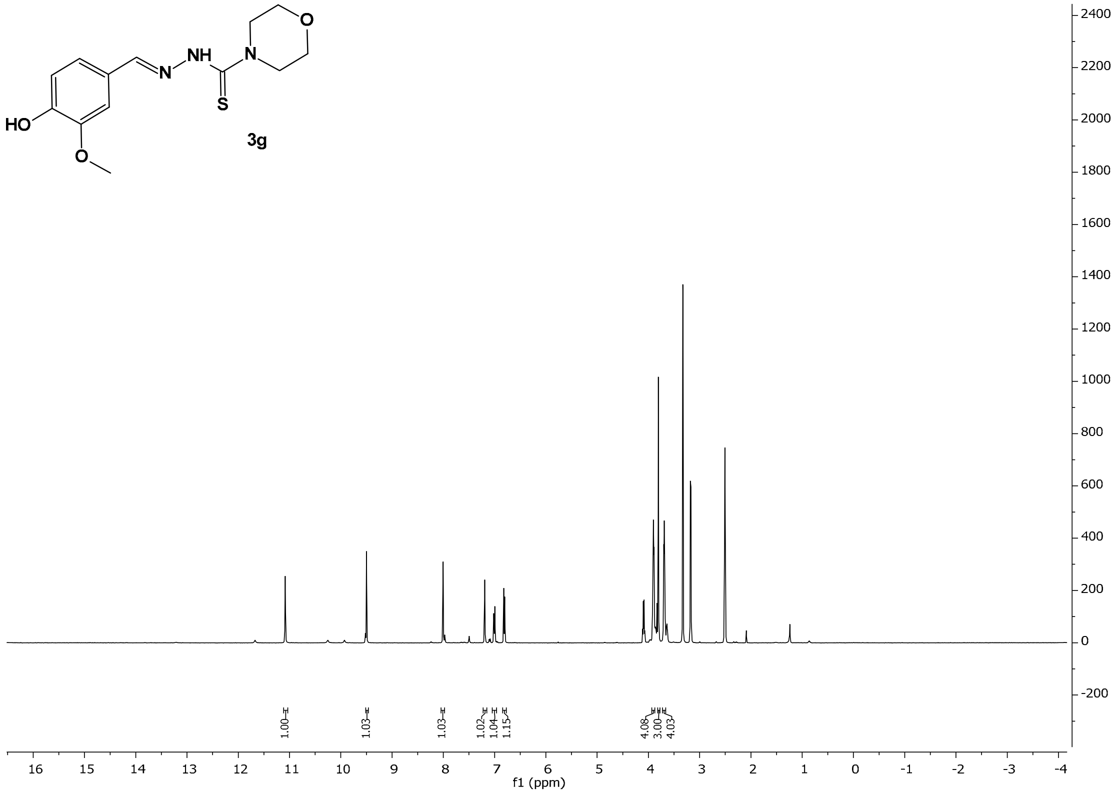

Figure S13. ^{1}H-NMR (DMSO-d_6) spectrum of *N*'-[(4-hydroxy-3-methoxyphenyl)methylidene]morpholine-4-carbothiohydrazide (**3g**).

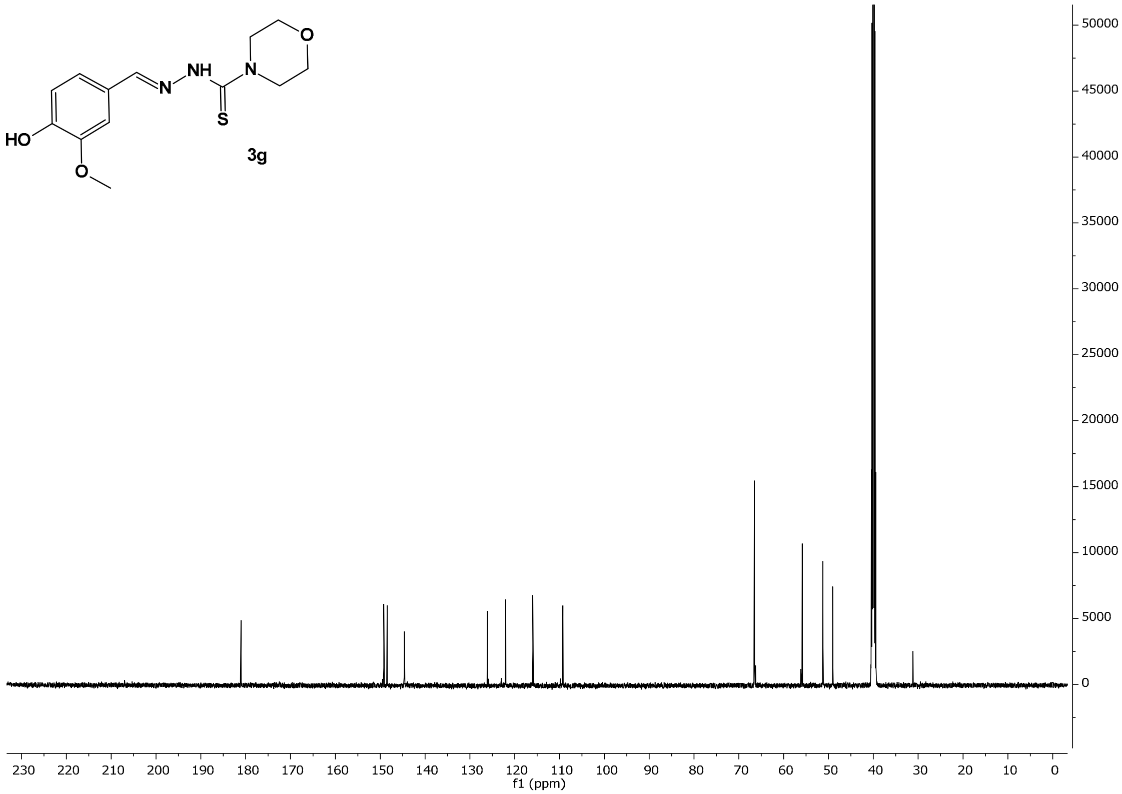

Figure S14. ^{13}C-NMR (DMSO-d_6) spectrum of *N*'-[(4-hydroxy-3-methoxyphenyl)methylidene]morpholine-4-carbothiohydrazide (**3g**).

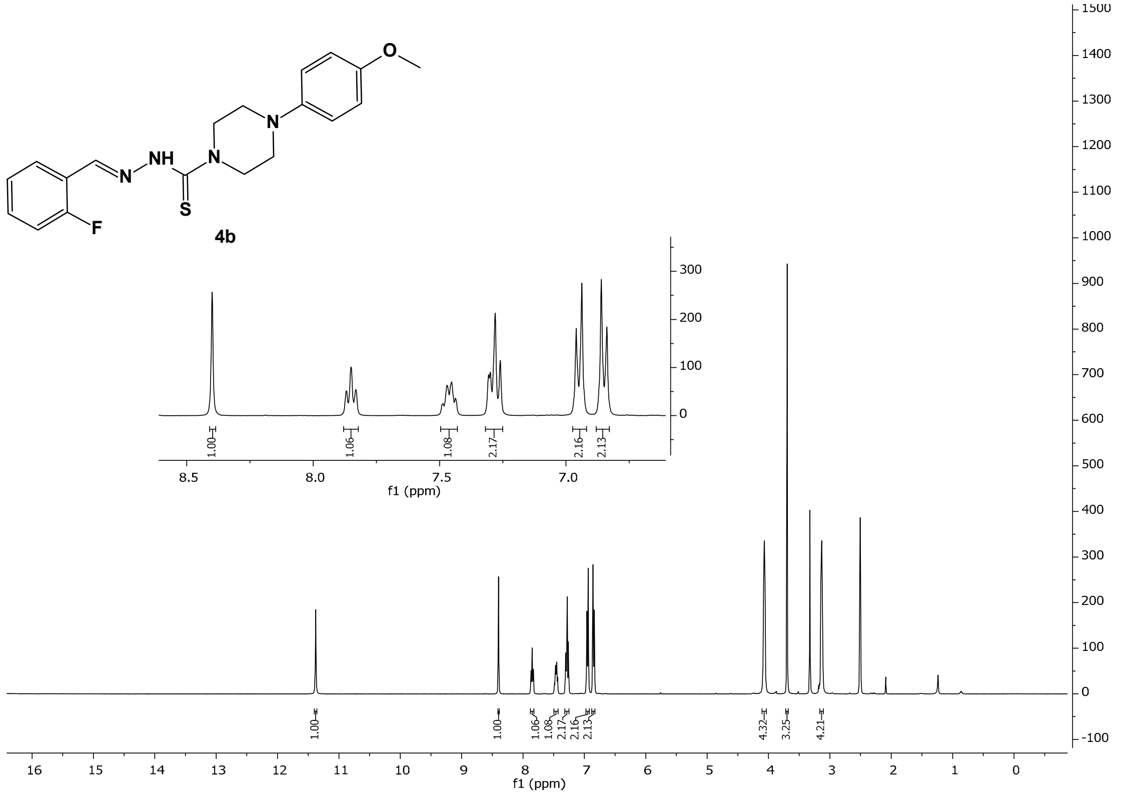

Figure S15. ^{1}H-NMR (DMSO-d_6) spectrum of *N'*-[(2-fluorophenyl)methylidene]-4-(4-methoxyphenyl)piperazine-1-carbothiohydrazide (**4b**).

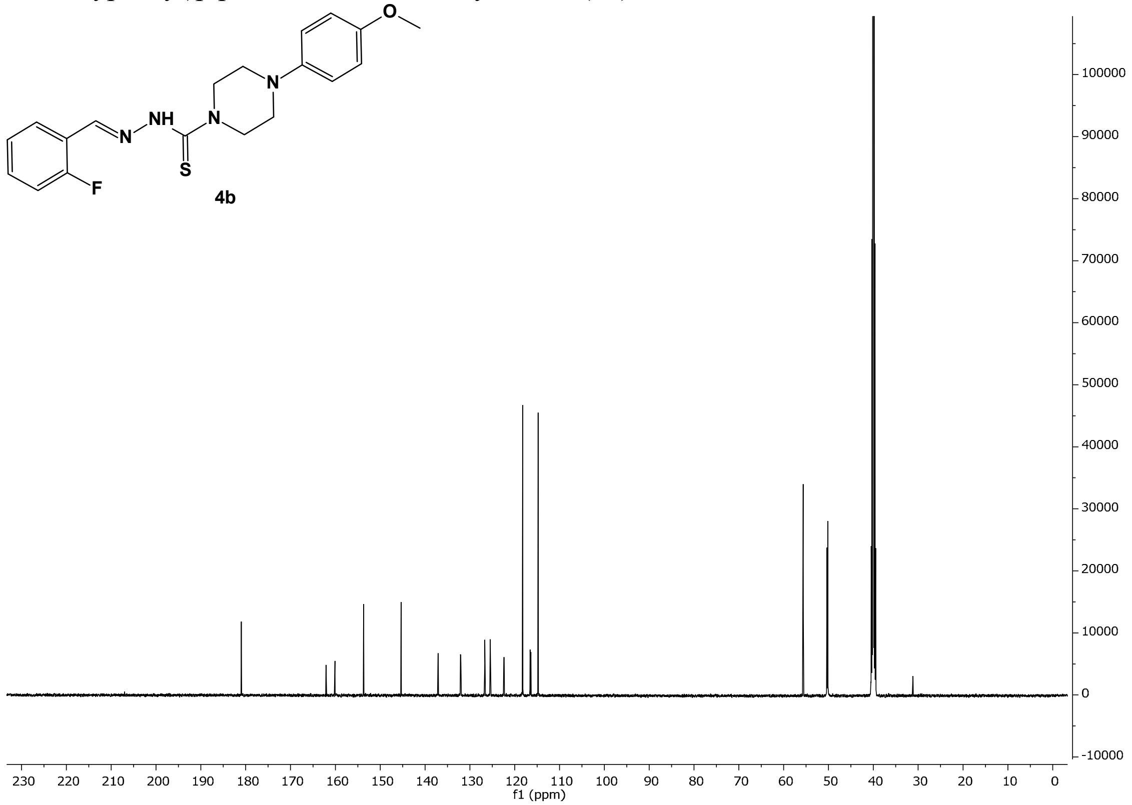

Figure S16. ^{13}C-NMR (DMSO-d_6) spectrum of *N'*-[(2-fluorophenyl)methylidene]-4-(4-methoxyphenyl)piperazine-1-carbothiohydrazide (**4b**).

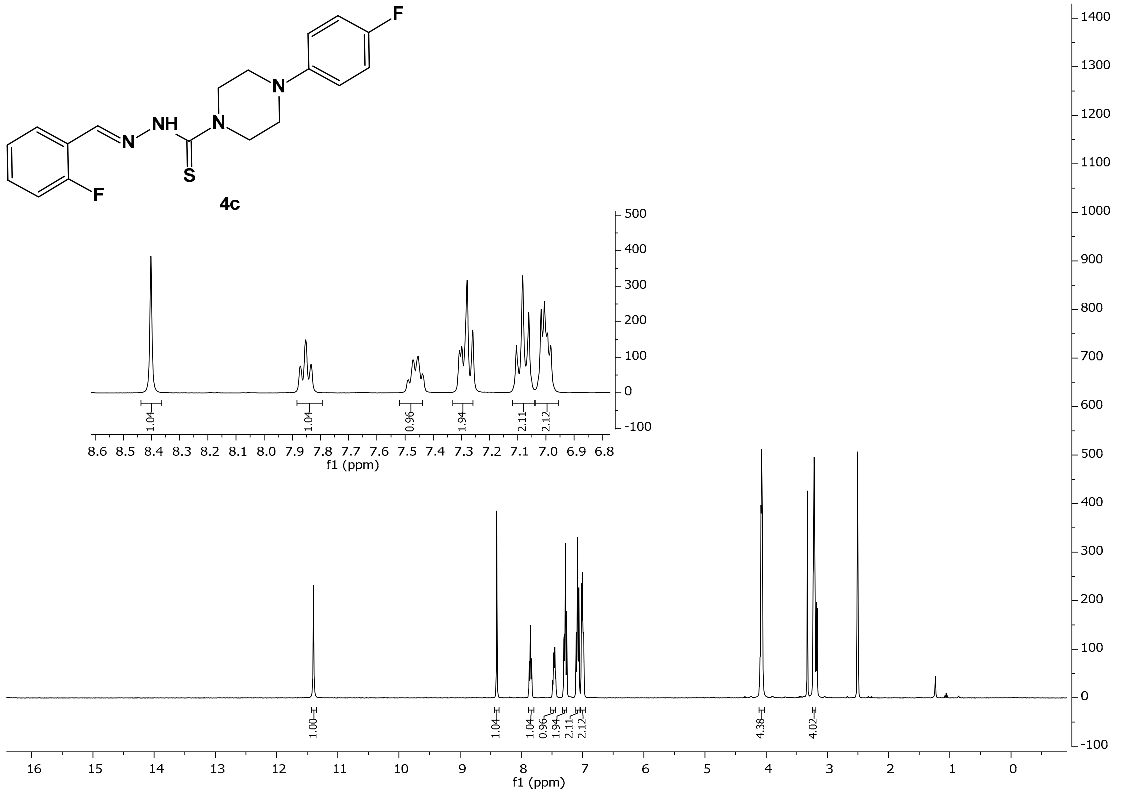

Figure S17. ^{1}H-NMR (DMSO-d_6) spectrum of *N'*-[(2-fluorophenyl)methylidene]-4-(4-fluorophenyl)piperazine-1-carbothiohydrazide (**4c**).

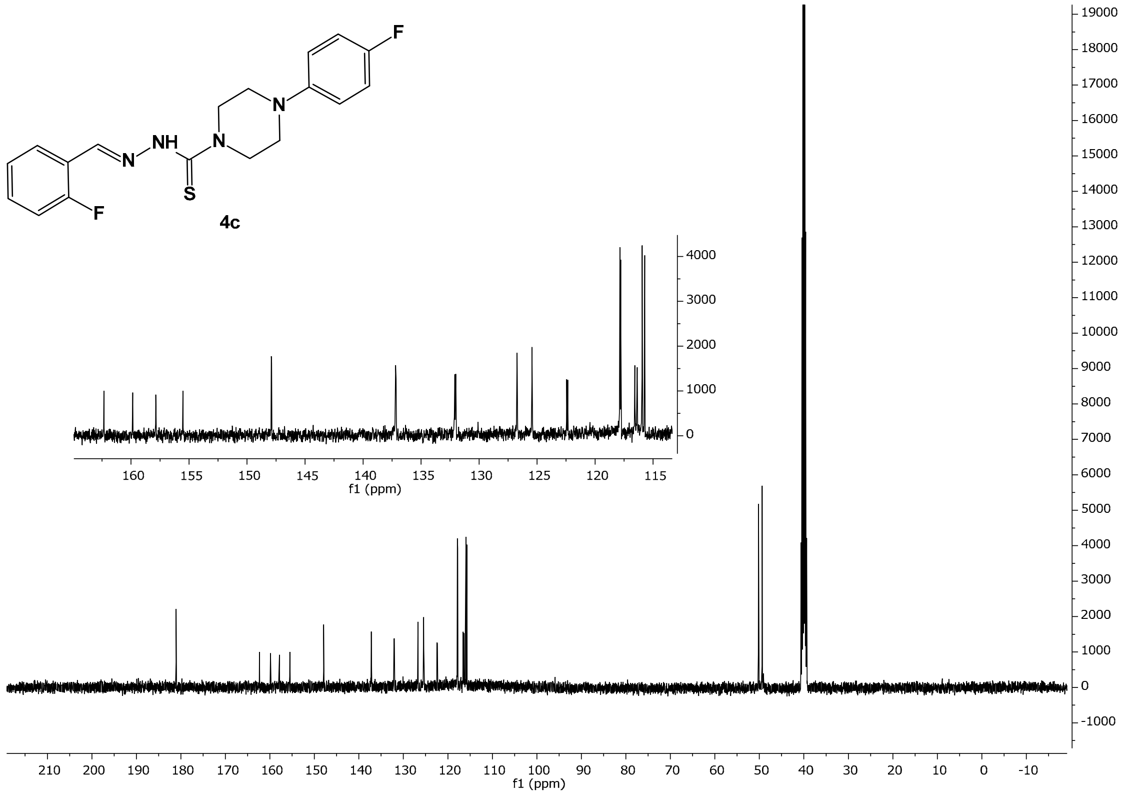

Figure S18. ^{13}C-NMR (DMSO-d_6) spectrum of *N'*-[(2-fluorophenyl)methylidene]-4-(4-fluorophenyl)piperazine-1-carbothiohydrazide (**4c**).

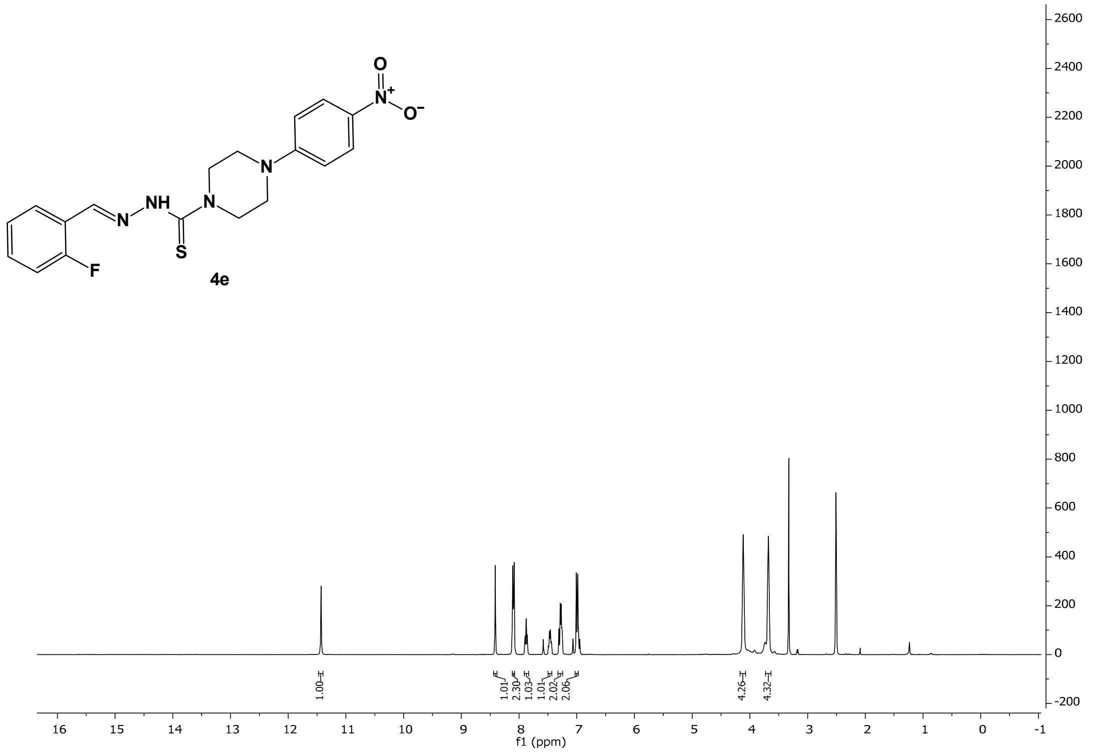

Figure S19. ^{1}H-NMR (DMSO-d_6) spectrum of *N'*-[(2-fluorophenyl)methylidene]-4-(4-nitrophenyl)piperazine-1-carbothiohydrazide (**4e**).

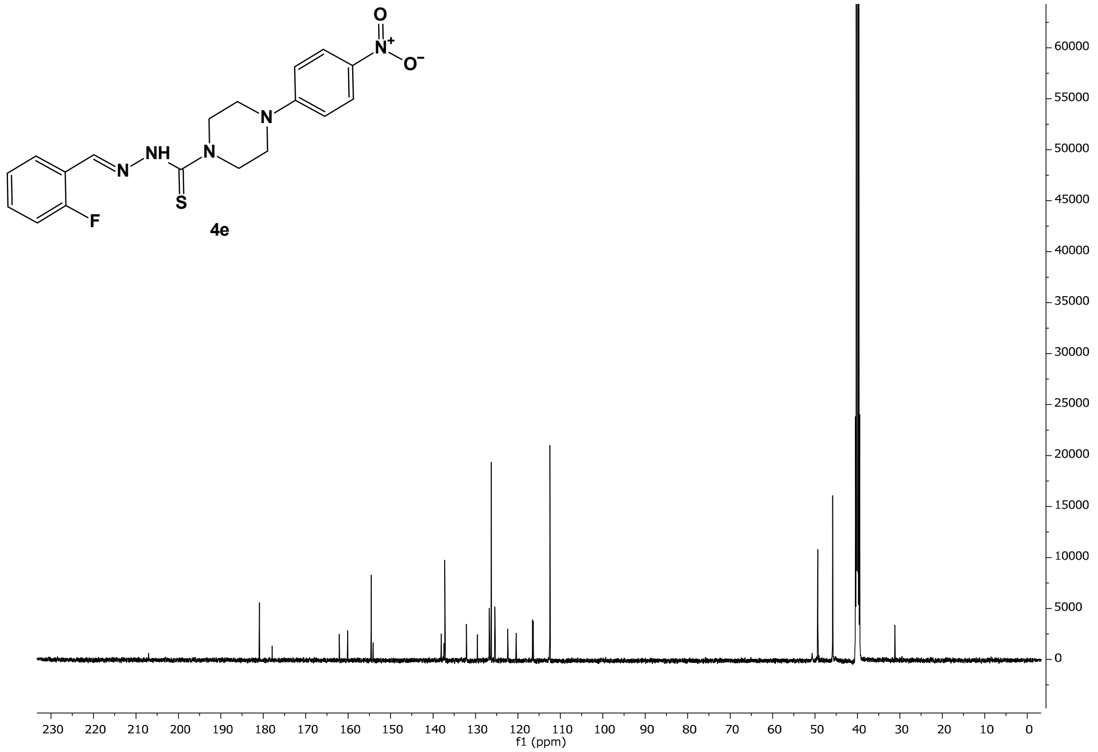

Figure S20. ^{13}C-NMR (DMSO-d_6) spectrum of *N'*-[(2-fluorophenyl)methylidene]-4-(4-nitrophenyl)piperazine-1-carbothiohydrazide (**4e**).

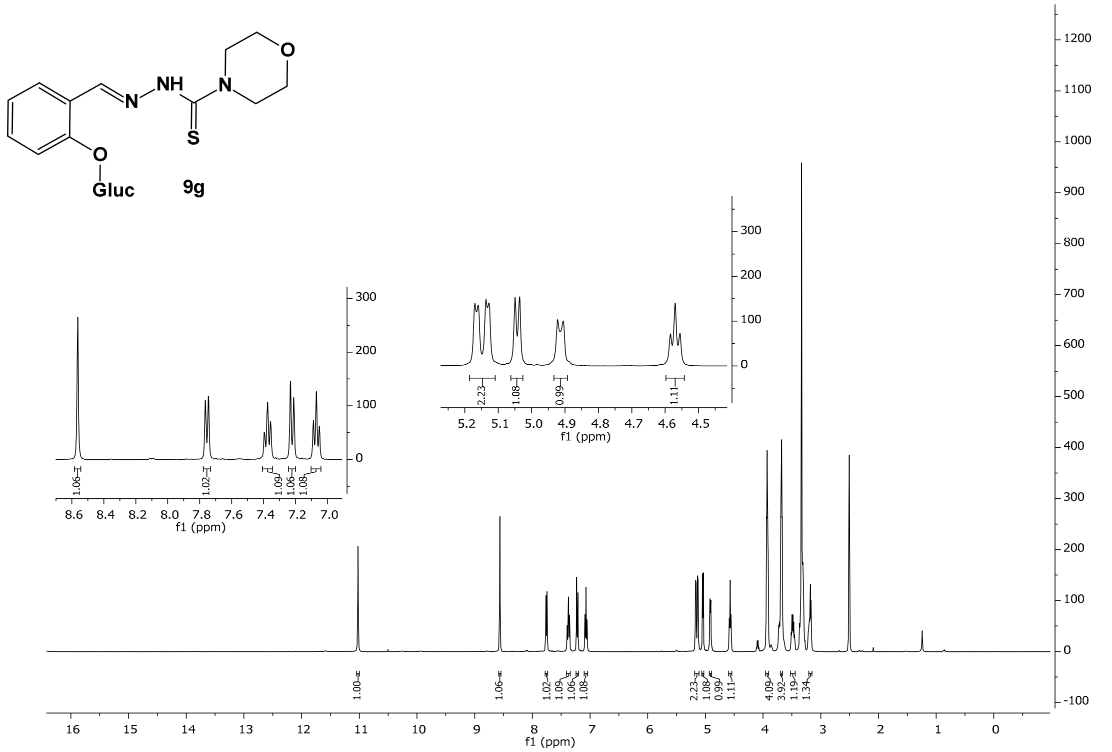

Figure S21. ^{1}H-NMR (DMSO-d_6) spectrum of *N*'-2{[(3,4,5-trihydroxy-6-hydroxymethylene)tetrahydropyran-2-yloxy]benzylidene}morpholine-4-carbothiohydrazide (**9g**).

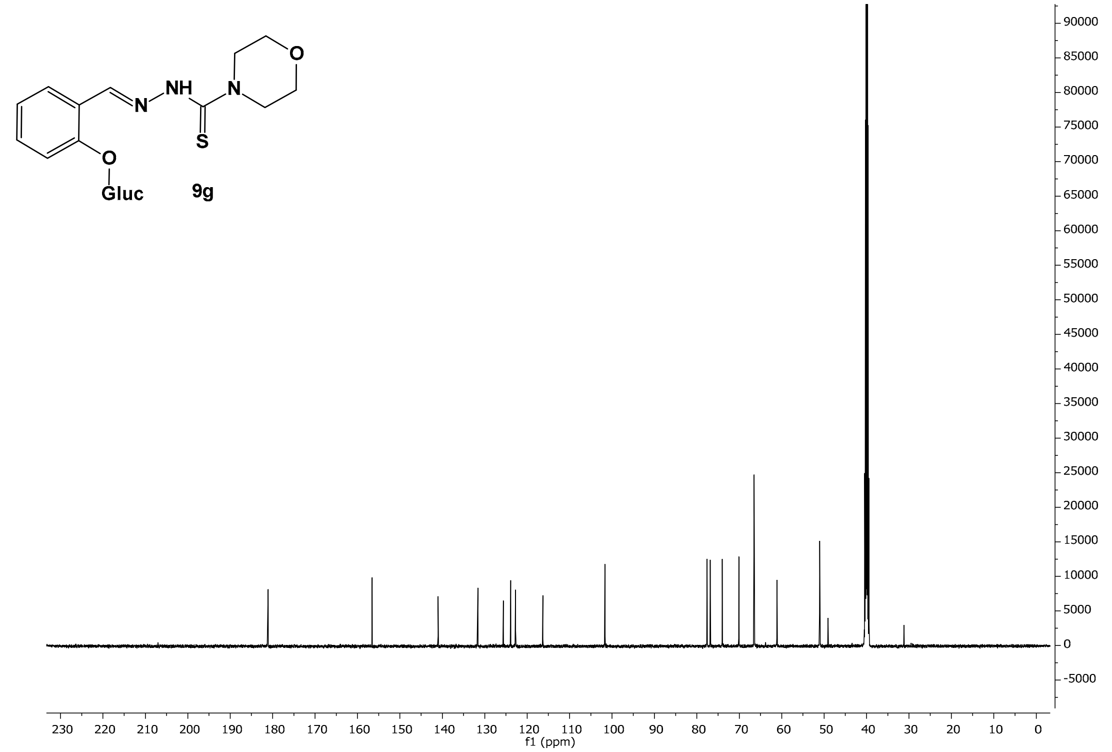

Figure S22. ^{13}C-NMR (DMSO-d_6) spectrum of *N*'-2{[(3,4,5-trihydroxy-6-hydroxymethylene)tetrahydropyran-2-yloxy]benzylidene}morpholine-4-carbothiohydrazide (**9g**).

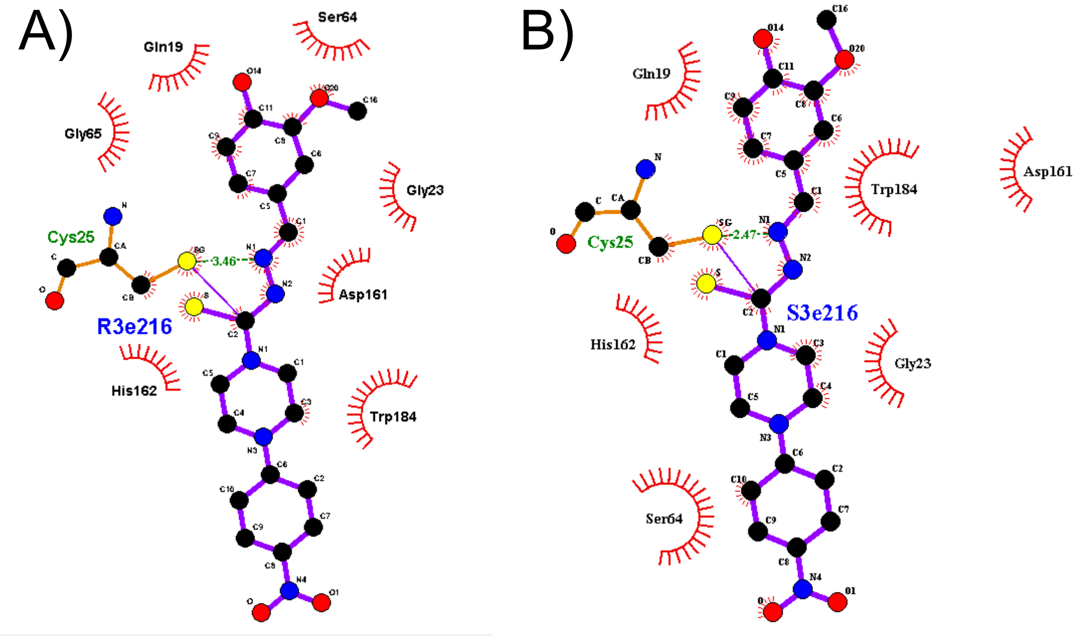

Figure S23. Interaction diagrams for compound **3e** (Panels A and B). Hydrogen bonds are represented as green dashed lines with the interatomic distance (Å) annotated, and nonbonded hydrophobic contacts are shown as red spoked arcs.

(A) **3e in *R*-pose.** The ligand forms a covalent adduct with Cys25, depicted as a direct bond between the Cys25 sulfur atom (SG) and the ligand (ligand atom labels as in the LigPlot+ output). A single, comparatively long SG···N2 interaction is annotated at 3.46 Å, consistent with a weak polar contact at the plotted geometry. The ligand is further surrounded by nonbonded contacts involving Gln19, Gly65, Gly23, Ser64, Asp161, His162, and Trp184, as indicated by the red spoked arcs (residue numbering as in the receptor model).

(B) **3e in *S*-pose.** The ligand forms a covalent adduct with Cys25 (SG). In this configuration, the SG···N2 hydrogen-bond annotation is shorter (2.47 Å), consistent with a more favorable hydrogen-bonding geometry than in Panel A. The surrounding nonbonded contact pattern is broadly similar and includes Gln19, Gly23, Ser64, Asp161, His162, and Trp184; Gly65 is not shown as a contacting residue in this panel, indicating a local reorientation of the ligand relative to Panel A.

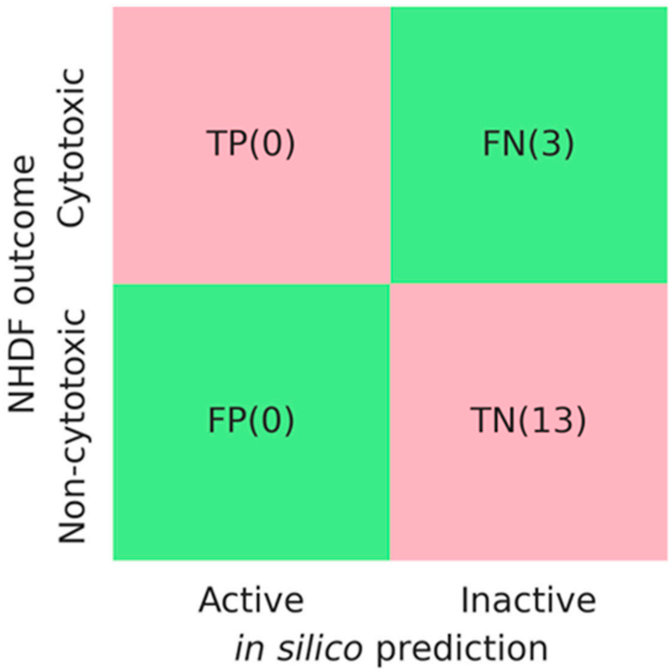

Figure S24. Heatmap benchmark of *in silico* predictions against experimental NHDF cytotoxicity, showing a confusion matrix populated only with true negatives (TN) and false negatives (FN); no true positives (TP) or false positives (FP) were observed.

Table S1. Tanimoto similarity matrix between each pair of piperazinyl thiosemicarbazone derivatives **2-10**. The binary Extended Connectivity Fingerprints (radius = 2, bit = 1024) was used for the similarity calculation.

	**2a**	**2b**	**2c**	**2d**	**2e**	**2f**	**2g**	**3b**	**3e**	**3f**	**3g**	**4b**	**4c**	**4e**	**5a**	**6c**	**7b**	**8a**	**9g**	**10e**
**2a**	1.000	0.719	0.759	0.683	0.724	0.755	0.648	0.485	0.465	0.477	0.388	0.455	0.492	0.463	0.774	0.550	0.414	0.562	0.321	0.305
**2b**	0.719	1.000	0.759	0.683	0.695	0.755	0.648	0.69	0.529	0.574	0.476	0.655	0.492	0.441	0.541	0.550	0.571	0.408	0.338	0.289
**2c**	0.759	0.759	1.000	0.719	0.732	0.800	0.686	0.508	0.464	0.500	0.406	0.525	0.660	0.508	0.569	0.731	0.412	0.406	0.351	0.300
**2d**	0.683	0.683	0.719	1.000	0.661	0.714	0.614	0.464	0.427	0.456	0.371	0.435	0.469	0.423	0.516	0.524	0.378	0.373	0.325	0.279
**2e**	0.724	0.695	0.732	0.661	1.000	0.727	0.625	0.471	0.656	0.463	0.377	0.441	0.476	0.667	0.548	0.532	0.603	0.397	0.312	0.453
**2f**	0.755	0.755	0.80	0.714	0.727	1.000	0.714	0.525	0.478	0.625	0.443	0.517	0.564	0.500	0.618	0.571	0.424	0.439	0.361	0.325
**2g**	0.648	0.648	0.686	0.614	0.625	0.714	1.000	0.413	0.377	0.426	0.585	0.381	0.414	0.369	0.466	0.474	0.324	0.319	0.446	0.225
**3b**	0.485	0.690	0.508	0.464	0.471	0.525	0.413	1.000	0.763	0.846	0.722	0.600	0.444	0.400	0.469	0.500	0.650	0.408	0.321	0.289
**3e**	0.465	0.529	0.464	0.427	0.656	0.478	0.377	0.763	1.000	0.759	0.650	0.457	0.406	0.576	0.449	0.456	0.750	0.377	0.298	0.430
**3f**	0.477	0.574	0.500	0.456	0.463	0.625	0.426	0.846	0.759	1.000	0.750	0.541	0.483	0.433	0.508	0.492	0.540	0.420	0.347	0.312
**3g**	0.388	0.476	0.406	0.371	0.377	0.443	0.585	0.722	0.650	0.75	1.000	0.4	0.344	0.31	0.369	0.397	0.446	0.301	0.42	0.214
**4b**	0.455	0.655	0.525	0.435	0.441	0.517	0.381	0.600	0.457	0.541	0.400	1.000	0.78	0.684	0.559	0.542	0.59	0.463	0.423	0.329
**4c**	0.492	0.492	0.660	0.469	0.476	0.564	0.414	0.444	0.406	0.483	0.344	0.78	1.000	0.75	0.611	0.686	0.438	0.476	0.455	0.351
**4e**	0.463	0.441	0.508	0.423	0.667	0.500	0.369	0.400	0.576	0.433	0.310	0.684	0.750	1.000	0.567	0.525	0.623	0.449	0.392	0.507
**5a**	0.774	0.541	0.569	0.516	0.548	0.618	0.466	0.469	0.449	0.508	0.369	0.559	0.611	0.567	1.000	0.589	0.439	0.655	0.394	0.338
**6c**	0.550	0.550	0.731	0.524	0.532	0.571	0.474	0.500	0.456	0.492	0.397	0.542	0.686	0.525	0.589	1.000	0.424	0.462	0.361	0.325
**7b**	0.414	0.571	0.412	0.378	0.603	0.424	0.324	0.65	0.750	0.540	0.446	0.590	0.438	0.623	0.439	0.424	1.000	0.423	0.300	0.440
**8a**	0.562	0.408	0.406	0.373	0.397	0.439	0.319	0.408	0.377	0.420	0.301	0.463	0.476	0.449	0.655	0.462	0.423	1.000	0.312	0.313
**9g**	0.321	0.338	0.351	0.325	0.312	0.361	0.446	0.321	0.298	0.347	0.420	0.423	0.455	0.392	0.394	0.361	0.300	0.312	1.000	0.231
**10e**	0.305	0.289	0.300	0.279	0.453	0.325	0.225	0.289	0.430	0.312	0.214	0.329	0.351	0.507	0.338	0.325	0.440	0.313	0.231	1.000
